# A lateral protrusion latticework connects neuroepithelial cells and is regulated during neurogenesis

**DOI:** 10.1242/jcs.259897

**Published:** 2022-03-30

**Authors:** Ioannis Kasioulis, Alwyn Dady, John James, Alan Prescott, Pamela A. Halley, Kate G. Storey

**Affiliations:** Division of Cell and Developmental Biology, School of Life Sciences, University of Dundee, Dundee DD1 5EH, UK

**Keywords:** Developing neuroepithelium, Apical endfeet, Cell protrusions, Actin dynamics, Live imaging

## Abstract

Dynamic contacts between cells within the developing neuroepithelium are poorly understood but play important roles in cell and tissue morphology and cell signalling. Here, using live-cell imaging and electron microscopy we reveal multiple protrusive structures in neuroepithelial apical endfeet of the chick embryonic spinal cord, including sub-apical protrusions that extend laterally within the tissue, and observe similar structures in human neuroepithelium. We characterise the dynamics, shape and cytoskeleton of these lateral protrusions and distinguish them from cytonemes, filopodia and tunnelling nanotubes. We demonstrate that lateral protrusions form a latticework of membrane contacts between non-adjacent cells, depend on actin but not microtubule dynamics, and provide a lamellipodial-like platform for further extending fine actin-dependent filipodia. We find that lateral protrusions depend on the actin-binding protein WAVE1 (also known as WASF1): misexpression of mutant WAVE1 attenuated protrusion and generated a round-ended apical endfoot morphology. However, this did not alter apico-basal cell polarity or tissue integrity. During normal neuronal delamination, lateral protrusions were withdrawn, but precocious protrusion loss induced by mutant WAVE1 was insufficient to trigger neurogenesis. This study uncovers a new form of cell–cell contact within the developing neuroepithelium, regulation of which prefigures neuronal delamination.

This article has an associated First Person interview with the first author of the paper.

## Abstract (precis)

**Summary:** Dynamic lateral protrusions from neuroepithelial apical endfeet form a lattice that connects non-neighbouring cells, is regulated during neurogenesis and may provide a basis for signalling across epithelia.

## INTRODUCTION

The patterning and production of differentiating cells within a proliferative epithelium is a central activity in tissue homeostasis and embryonic development. Recent advances have identified roles for cellular protrusions in these processes in a range of tissues including epithelia (Gerdes and Pepperkok, 2013; González-Méndez et al., 2019; Mattes and Scholpp, 2018). It is now clear that there are many types of cellular protrusion and that these vary in dimension, dynamics and involvement of specific cytoskeletal components. Such protrusions can also be distinguished by whether their protrusive behaviour results in cell-to-cell membrane contact, as exhibited by cytonemes and filopodia (Ramírez-Weber and Kornberg, 1999), or connection and the sharing of cytoplasmic content including small organelles, which is characteristic of tunnelling nanotubes (Rustom et al., 2004). Cytonemes are long, fine, actin-dependent filopodia that can contact cells up to several hundred micrometres away and were first shown to mediate signalling at a distance in *Drosophila* (Cohen et al., 2010; De Joussineau et al., 2003; Hsiung et al., 2005; Rajan et al., 2009). Since then, a plethora of similar cellular protrusions, some containing microtubules at their base, have been described in other embryos, and their functional significance is beginning to be elucidated (González-Méndez et al., 2019). In the developing vertebrate nervous system, this has been most closely addressed in zebrafish, where fine actin-dependent filopodia deliver Wnt ligand to pattern the neural plate (Luz et al., 2014; Mattes et al., 2018; Stanganello et al., 2015). Later, in the neural tube, the Notch ligand Delta is presented by transient basally originating cell protrusions produced by newborn neurons to regulate the spatial and temporal production of spinal cord neurons (Hadjivasiliou et al., 2019, 2016). A correlation between filopodia and Notch ligand presentation has also been identified in the mouse cortex (Moore et al., 2020; Nelson et al., 2013), whereas other work supports a dependence on apical adherens junction-localised Notch ligands in chick and mouse neuroepithelium (Hatakeyama et al., 2014). The former findings with respect to Wnt signalling, and others reporting presentation of further ligands or their receptors, including sonic hedgehog, bone morphogenetic proteins and fibroblast growth factors, in non-neural contexts (Huang et al., 2019; Roy et al., 2014; Sanders et al., 2013) raise an alternative or complementary hypothesis to the notion that signal diffusion through extracellular space underlies establishment of signalling gradients that pattern developing tissues (Briscoe and Small, 2015; Kornberg, 2014; Wolpert, 2016). Whether similar cell protrusions exist and mediate patterning and neuronal differentiation or confer mechanical properties in the developing neuroepithelium in other model vertebrates or in human tissue is yet to be determined.

In the adult nervous system, cellular protrusions have been proposed to mediate pathogen spread in the form of prions and to facilitate progression of neurodegenerative diseases such as Alzheimer's (Victoria and Zurzolo, 2017). A further related possibility is that cell protrusions propagate infectious agents within the developing neuroepithelium. These considerations highlight the importance of elucidating protrusive cell behaviour, particularly at the apical surface of the neuroepithelium, which is exposed to the fluid-filled ventricle of the neural tube. The apical surface is made by the apical endfeet of neuroepithelial cells, which are linked together by adherens junctions containing N-cadherin (also known as CDH2) and are essential for cell adhesion and tissue integrity (Hatta and Takeichi, 1986; Kadowaki et al., 2007; Meng and Takeichi, 2009). The pseudostratified nature of the neuroepithelium is characterised by interkinetic nuclear migration as neural progenitors progress through the cell cycle and divide at the apical surface (Miyata, 2008; Sauer, 1935). Within this proliferating cell population, individual cells are then selected for neuronal differentiation by rising proneural gene expression and lateral inhibition mediated by Notch–Delta signalling (Henrique et al., 1995; Henrique and Schweisguth, 2019). Importantly, the transcription factor cascade that drives neuronal differentiation regulates the expression of genes encoding cadherins (Itoh et al., 2013; Rousso et al., 2012), reduction of which impacts adherens junctions and the linked intracellular actin network in the apical endfoot. These steps prefigure the delamination of newborn neurons from the ventricular surface in a process that involves local actin and microtubule cytoskeletal reconfiguration (Camargo Ortega et al., 2019; Kasioulis and Storey, 2018; Kawaguchi, 2020) and, in the chick and mouse neural tube, abscission of the apical membrane and dismantling of primary cilia (Das and Storey, 2014; Kasioulis et al., 2017; reviewed in Kasioulis and Storey, 2018; Paridaen and Huttner, 2014). The presence of apical microvilli has been documented using electron microscopy (Shepard et al., 1997, 1993; Smith et al., 1982; Waterman, 1976) and immunofluorescence (Marzesco et al., 2005; Weigmann et al., 1997). Recent analyses have also identified sub-apical protrusions implicated in tissue elasticity underlying interkinetic nuclear migration (Shinoda et al., 2018). However, detailed characterisation of the range of protrusive structures at the apical surface and extending sub-apically within the vertebrate neuroepithelium using live-imaging approaches has yet to be undertaken.

Here, we take advantage of the ability to transfect a mosaic of neuroepithelial cells in the chicken embryonic spinal cord to live image and monitor apical and sub-apical protrusion dimensions and dynamics. We further use electron microscopy to document apical endfoot protrusions in chicken and human neuroepithelium. Focusing on lamellipodia-like sub-apical lateral protrusions, we parameterise these structures and demonstrate that they contact protrusions from non-neighbouring cells. Moreover, sub-apical lateral protrusions provided a platform for extension of fine filopodia, which we distinguish from cytonemes and tunnelling nanotubes, and so extend contact over several apical endfoot diameters. Distinct configurations of actin networks are regulated by distinct actin-nucleating proteins of the WAVE family (Campellone and Welch, 2010; Sweeney et al., 2015; Yamazaki et al., 2005), and we show here that sub-apical lateral protrusions depend on WAVE1 (also known as WASF1). We investigate the functional consequences of WAVE1 manipulation and implicate regulation of the WAVE1-mediated actin network in neuronal delamination.

## RESULTS

### Multiple protrusive structures emerge from neuroepithelial apical endfeet

**Fig. 1.**
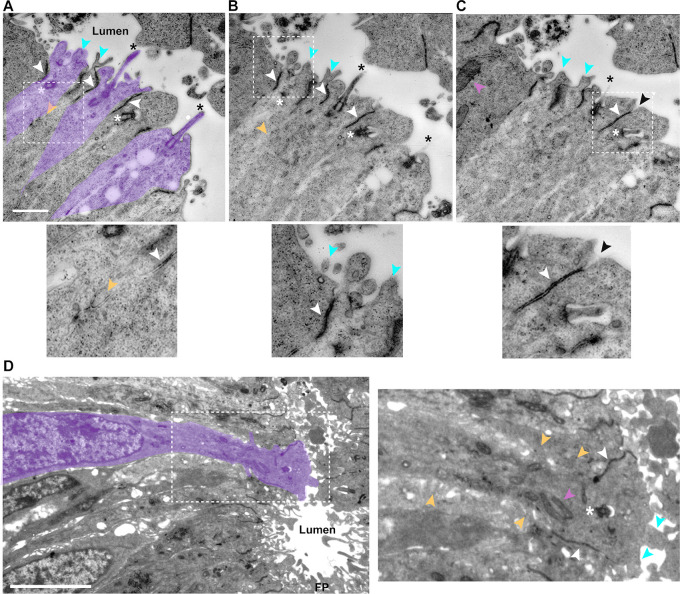
**Ultrastructure of chicken and human embryo neuroepithelial apical endfeet.** (A–C) Serial transverse sections of chicken embryonic spinal cord. In A, alternate endfeet are indicated in purple (excluding adherens junctions). White dashed boxes indicate regions enlarged below each image. (D) Transverse section through human embryonic spinal cord (CS12) in the ventral region, including floorplate (FP). The region marked by the white dashed box is enlarged in the right-hand image. Black asterisks, primary cilia tips in the lumen; basal bodies of primary cilia, white asterisks; adherens junctions (identified by electron-dense material near abutting membranes), white arrowheads; microvilli, blue arrowheads; lateral protrusions, yellow arrowheads; mitochondria, pink arrowheads. Scale bars: 1 μm (A–C), 5 μm (D).

To investigate the ultrastructure of neuroepithelial apical endfeet we used transmission electron microscopy (TEM), generating thin transverse sections of spinal cord (interlimb region) from chicken [Hamburger and Hamilton (HH) stage 12] (Fig. 1A–C) and human [Carnegie stage (CS) 12] embryos (Fig. 1D). This revealed key features in serial sections of the chicken tissue, including lumen-protruding primary cilia (asterisks) and microvilli with a range of morphologies (blue arrowheads) (Fig. 1A–C). Focusing on contacts between neuroepithelial cells, we identified thin plasma membrane that spread between adjacent endfeet (Fig. 1C, black arrowheads) apical to adherens junctions (white arrowheads), and sub-apical membrane protrusions (yellow arrowheads) that extend laterally over neighbouring cells below adherens junctions. These key sub-cellular structures could also be identified in human tissue (Fig. 1D), which was distinguished by the presence of extensive microvilli, sub-apical lateral protrusions and prominent mitochondria (pink arrowheads).

To investigate the dynamics of protrusive structures in apical endfeet, the neural tube of HH10–12 chick embryos was transfected with a plasmid encoding the membrane marker pm-eGFP, and the embryos were then cultured in ovo to stages HH17–18. This approach labelled cells in a mosaic fashion and allowed rapid live imaging of the membrane dynamics of individual endfeet from the apical surface (en face) and up to 5 μm basally into tissue explants.

**Fig. 2.**
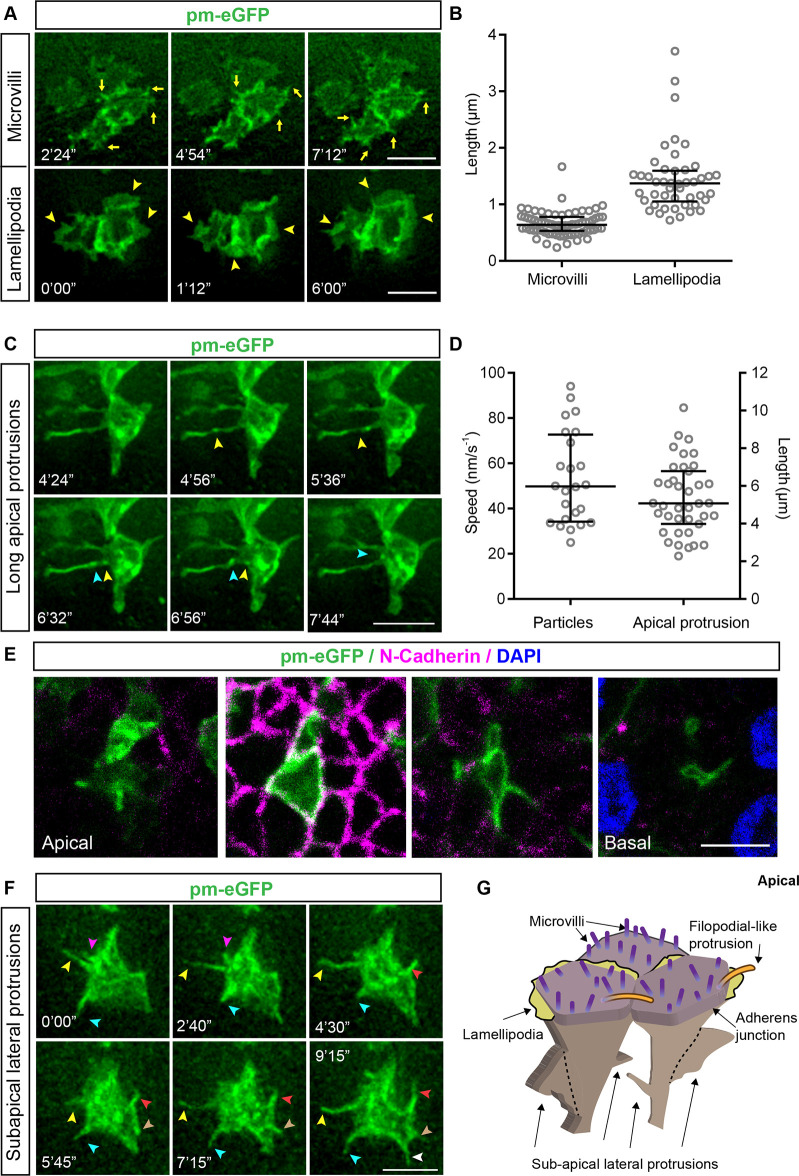
**Live-imaging analysis of apical and sub-apical protrusions extending from neuroepithelial apical endfeet.** (A) En face live imaging of neuroepithelial cell apical endfeet expressing the membrane marker pm-eGFP. Representative timepoints showing apical microvilli (yellow arrows) and lamellipodia (yellow arrowheads) at the apical surface. (B) Quantification of microvilli and lamellipodial length for cells as in A (microvilli: three experiments, seven explants, 28 cells, 70 microvilli; lamellipodia: three experiments, seven explants, 46 cells). (C) Representative timepoints of en face live imaging, as described in A, showing long thin apical protrusions and/or filopodia extending into the neural tube lumen (see Movie 4). (D) Quantification of surface protrusion length (right) and speed of particle movement along the long thin apical protrusions (left). Nine experiments, 14 explants, 39 cells. (E) Serial *z* stack images of neuroepithelial cell apical endfeet expressing the membrane marker pm-eGFP (left to right, apical to basal), showing sub-apical lateral protrusions emerging below N-cadherin-expressing adherens junctions (pink). Nuclei are stained with DAPI. (F) Representative timepoints showing sub-apical lateral protrusions. Coloured arrowheads follow the dynamics of different sub-apical protrusions over time. (G) Summary schematic of apical endfoot protrusions. Microvilli and microspikes, lamellipodia and long protrusions form at the apical surface (note primary cilia are not represented here). Sub-apical lateral protrusions emerge below the adherens junctions. Error bars in B and D show the median and interquartile range. Scale bars: 5 μm.

A variety of apical membrane protrusions were observed, and these could be classified based on apico-basal position, size and dynamics. At the apical-most (lumen-facing) surface, short microvilli (also known as microspikes; 0.66±0.21 μm, mean±s.d.) (Shepard et al., 1997, 1993) consistent with those identified by TEM were most apparent, along with novel lamellipodia-like protrusive structures (1.44±0.60 μm) that may correspond to the thin plasma membrane observed between endfeet by TEM; microspikes and these apical lamellipodia-like protrusions (hereafter referred to as lamellopdia) were found in both live and fixed tissue (Fig. 2A,B). Monitoring F-tractin localisation further confirmed that both the lamellipodia and the microvilli protrusions into the ventricle were actin rich (Movies 1–3). In addition, longer thin protrusions (5.34±1.89 μm) that extended into the lumen and across neighbouring endfeet were observed at this apical-most surface. These filopodial-like protrusions were not apparent in our TEM analysis but have been reported previously in SEM analyses (Shepard et al., 1997, 1993). Strikingly, these structures uniquely exhibited retrograde membrane movements (20–90 nm/s^−1^), consistent with the possibility of protein trafficking from the luminal space (Fig. 2C,D; Movie 4).

Using this live-imaging approach, a further set of membrane protrusions were observed, this time emerging sub-apically, beneath the N-cadherin-expressing adherens junction (Fig. 2E; Movie 5) and elongating lateral to the apico-basal axis, (Fig. 2F; Movie 6). These confirmed our TEM observation of sub-apical lateral protrusions: these are similar to protrusive structures recently described in the developing mouse cortex (Shinoda et al., 2018). In conclusion, the developing chick neuroepithelium contains at least three types of dynamic apical membrane protrusions (microvilli, lamellipodia and filipodia), in addition to primary cilia, and exhibits sub-apical laterally extending protrusions, which hereafter we will refer to as lateral protrusions (Fig. 2G).

### Characterisation of dynamic lateral protrusions within developing neuroepithelium

**Fig. 3.**
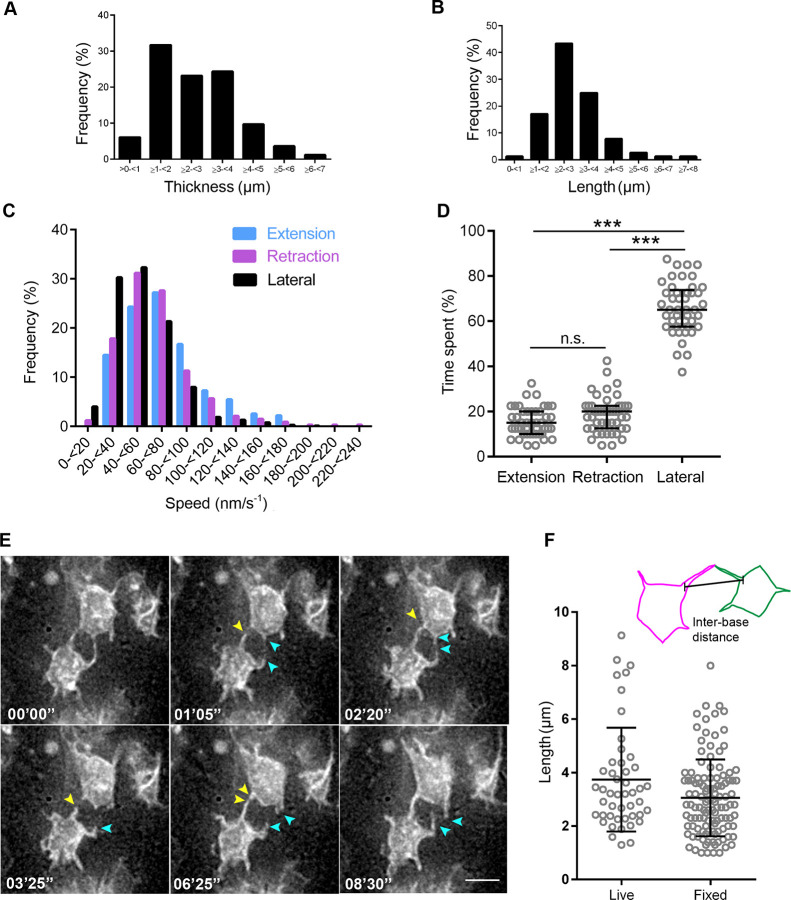
**Characterisation of lateral protrusions.** (A) Thickness of lateral protrusions and their frequency (three experiments, 5 explants, 82 cells). (B) Protrusion length in contacting pairs (five experiments, nine explants, 48 pairs). (C) Histogram of protrusion speed and frequency of extension, retraction, and lateral movement. (D) Quantification of time that individual protrusions extend, retract or undergo lateral movement. Total time analysed per protrusion is 10 min. Error bars show the median and interquartile range. ***P<0.0001; n.s., not significant, *P*=0.1786 (one-way ANOVA with Tukey's multiple comparisons test). (E) Timepoints from live imaging of lateral protrusions. Arrowheads follow the dynamics of protrusions over time and cells express the membrane marker pm-eGFP. Scale bar: 5 μm. (F) Quantification of lateral protrusion distance from their base, as indicated in the schematic (top), measured in fixed and live-imaged tissue (fixed: three experiments, seven explants, 116 cells; live: five experiments, nine explants, 47 cells). Error bars show the mean±s.d.

To characterise lateral protrusions, we misexpressed pm-eGFP alone or together with pm-mKate2 and live imaged these structures at short intervals to capture their dimensions (thickness and length) as well as speed and direction of movement. Measurement of individual lateral protrusions revealed these to be thick (2–4 μm across), short processes (most 2–3 μm in length, ranging up to 7 μm) (Fig. 3A,B). To visualise lateral protrusion shape, explants fixed at the end of live-imaging sessions were subjected to higher magnification imaging using confocal microscopy to generate 3D models of apical endfeet: these confirmed protrusion apico-basal position, thickness and length (Fig. 3A,B; Movie 7). This analysis also revealed that all cells observed had apical endfeet with lateral protrusions, including cells in mitosis (Fig. S1).

To analyse lateral protrusion dynamics, the direction of their movement was categorised as either extension, retraction or lateral. Extension and retraction speeds ranged between 40 and 80 nm/s^−1^, with a few more rapid examples with speeds of 160–180 nm/s^−1^ (Fig. 3C). In contrast, lateral movement was slower (20–60 nm/s^−1^) and was detected most of the time (Fig. 3D): this may reflect passive displacement influenced by shape changes of neighbouring cells within the tissue.

A striking feature of lateral protrusions, made apparent by the mosaic transfection of cells, is their extension around immediately neighbouring endfeet and contact with similar protrusions from more distant cells within the tissue (Fig. 3E; Movies 8, 9). We measured the direct distance between the base of protrusions that contacted each other and found that the majority were 1–5 μm apart, while a subset reached across 5–10 μm; similar findings were made in fixed tissue (live, 3.73±0.28 μm; fixed, 3.1±0.13 µm; mean±s.d.) (Fig. 3F). As apical endfeet range from ∼3 to 6 μm in diameter (Fig. S2A) and lateral protrusions extend ∼2–3 μm (Fig. 3B), this indicates that most lateral protrusions make contact with protrusions from non-neighbouring cells. The extent of protrusion–protrusion contacts between non-neighbouring cells was next measured by analysing image *z*-stacks. Most protrusion contacts ranged up to 2 μm (Fig. S2B).

**Fig. 4.**
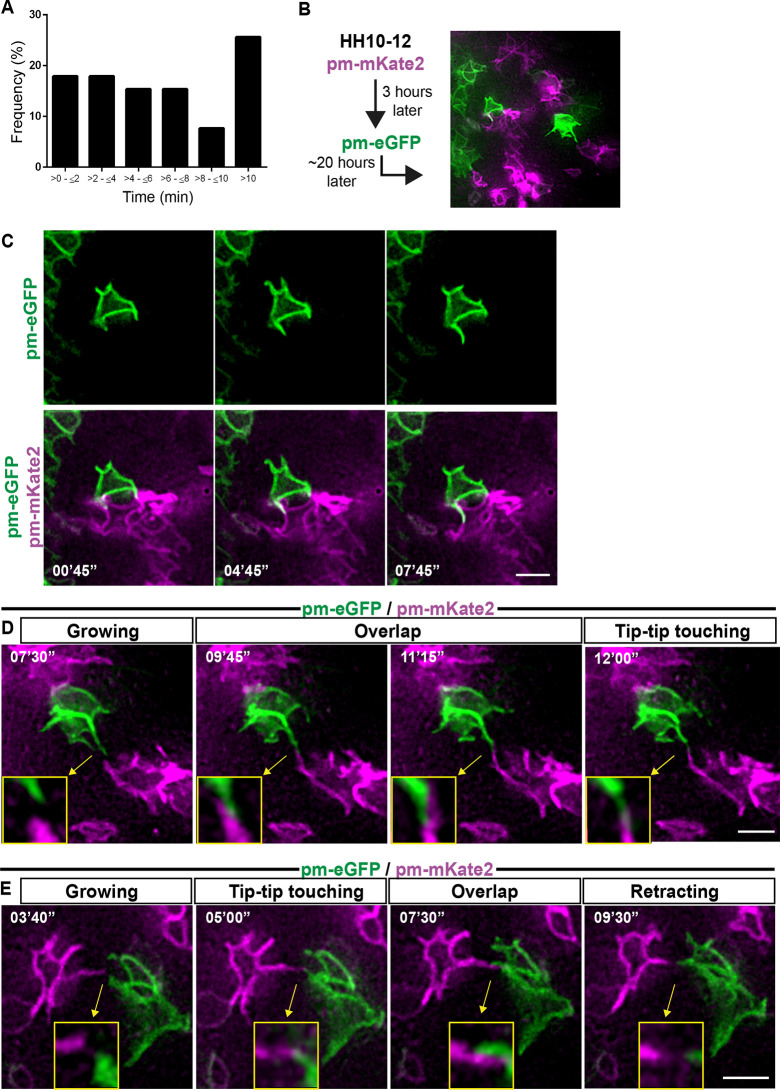
**Lateral protrusion contacts from non-neighbouring cells extend along each other and can be long-lived.** (A) Bar chart showing duration and frequency of protrusion contacts (five experiments, nine explants, 39 cell pairs). (B) Strategy for generating mosaic pattern of pm-eGFP- and pm-mKate2-expressing cells. Cells were first transfected with pm-mKate2 at HH10–12 and 3 h later with pm-EGFP. Embryos were then left to develop to stage HH17–18 for live imaging. (C) Representative timepoints showing that protrusions grow along the sides and edges of neighbouring cells. (D,E) Representative timepoints showing protrusions from non-neighbouring cells contacting each other at their tips and extending along each other. Arrows and insets show the contact region magnified. Scale bars: 5 μm.

In addition, it was clear that lateral protrusion length varied from cell to cell in contacting pairs (Fig. S2C). Importantly, these protrusion–protrusion contacts were dynamic and could persist for more than 10 min (longest contact time recorded 22 min 55 s; Fig. 4A). To investigate contact dynamics in detail we transfected the neuroepithelium with a mixture of the membrane markers pm-eGFP and pm-mKate2 (Fig. 4B), generating a mosaic of cells expressing either one of these fluorophore-tagged proteins. Live imaging confirmed lateral protrusion extension around the curvature of neighbouring endfeet (Fig. 4C; Movie 10) and revealed that protrusions from non-neighbouring cells meet tip to tip, extend along each other and also retract (Fig. 4D,E; Movies 11, 12). These behaviours suggest that lateral protrusions do not fuse but rather generate close membrane contacts between non-neighbouring cells.

Taken together, these observations identify a new form of cell–cell contact within the developing neuroepithelium mediated by a sub-apical latticework of laterally extending protrusions, which connect non-neighbouring cells.

### A unique cytoskeletal architecture distinguishes lateral protrusions

**Fig. 5.**
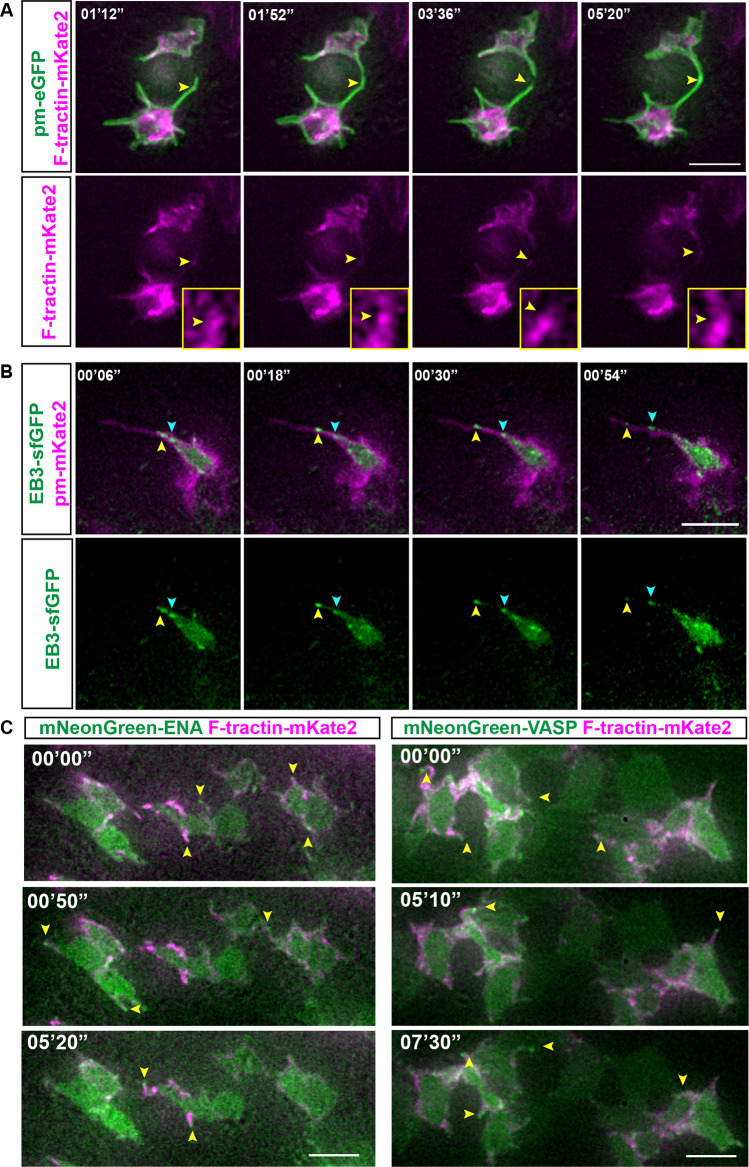
**Actin, microtubules and Ena/VASP proteins localise to lateral protrusions.** (A–C) Timepoints from live-imaging sessions. (A) F-tractin–mKate2 follows the dynamics of membrane protrusions. The membrane is marked by pm-eGFP. Yellow arrowheads indicate F-tractin–mKate2 dynamics (two experiments, six explants). Inset images show regions indicated by arrowheads. (B) Unidirectional microtubule movement from the base to the tip of the protrusion. Arrowheads follow the movement of EB3–sfGFP comets (two experiments, five explants). The membrane is marked by pm-mKate2. (C) ENA and VASP localising at the tip of protrusions (yellow arrowheads). Images are representative of three experiments and 10 explants. Scale bars: 5 μm.

To characterise the cytoskeletal architecture of lateral protrusions, plasmids expressing constructs that labelled actin (F-tractin–mKate2) or microtubule comets (EB3–sfGFP) were transfected along with membrane markers into the neural tube. F-tractin–mKate2 localisation followed the extension and retraction dynamics of the protrusions and was detected close to the protrusion tip (Fig. 5A; Movie 13). EB3–sfGFP comets showed a unidirectional movement from base to tip in most protrusions (Fig. 5B; Fig. S3A, Movie 14). Moreover, co-transfection of F-tractin–mKate2 and EB3–sfGFP showed that actin and microtubules elongate to the same extent within lateral protrusions (Fig. S3B,C).

Proteins involved in actin filament elongation in many different contexts include Ena proteins and vasodilator-stimulated phosphoprotein (VASP) (Dent et al., 2007; Gallop, 2020; Kwiatkowski et al., 2007; Lebrand et al., 2004). Ena/VASP proteins are also proposed to support junctional actin assembly in tissue that is under frequent tension of myosin-induced contractility, including at the level of adherens junctions (Leerberg et al., 2014). To investigate whether these proteins are localised to lateral protrusions, we misexpressed (as above) mNeonGreen fused to either ENA (mNeonGreen–ENA) or VASP (mNeonGreen–VASP) (both amplified from *Xenopus laevis*; known as ENAH and VASP in humans) together with the actin marker F-tractin–mKate2, and both proteins were found at adherens junctions (Fig. S3D,E; Movies 15, 16), and at lateral protrusion tips (Fig. 5C; Movies 17, 18). In contrast, a further tip localised regulator of filopodial elongation, unconventional myosin X [bovine myosin X (MYO10); Sanders et al., 2013], was detected uniformly in the cell cytoplasm but did not localise to lateral protrusion tips (Fig. S3F). However, following misexpression, myosin X was found at the tip of a separate set of longer dynamics protrusions, the origin of which was difficult to determine (Movie 19). Along with their shorter and broader dimensions, these further findings distinguish lateral protrusions from elongating filopodia, which exhibit prominent tip-localised myosin X, and, consistent with their expression of Ena/VASP proteins (Damiano-Guercio et al., 2020), indicate that lateral protrusions have lamellipodia-like characteristics.

**Fig. 6.**
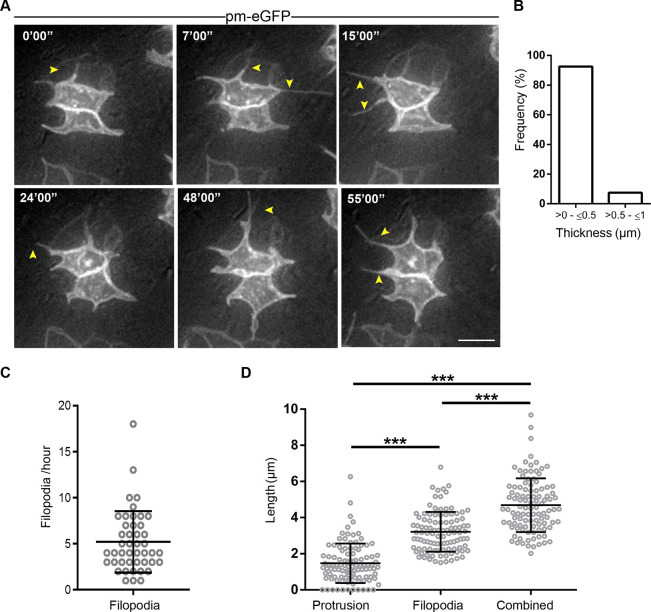
**Filopodia emerge from lateral protrusions.** (A) Live-imaging timepoints of cells expressing pm-eGFP, showing presence of thin filopodial extensions (yellow arrowheads) emerging from lateral protrusions. Scale bar: 5 μm. (B) Bar chart showing that filopodial protrusions are ∼<0.5 μm in thickness (three experiments, five explants, 65 filopodia). (C) Dot plot showing number of filopodia formed in 1 h (5.2±3.3 per hour, mean±s.d.; four experiments, seven explants, 45 cells). (D) Filopodia are significantly longer than lateral protrusions, and the combined filopodium–protrusion length extends lateral reach (three experiments, five explants, 27 cells). ***P<0.0001 (one-way ANOVA with Tukey's multiple comparisons test). Error bars in C and D show mean±s.d.

To monitor lateral protrusion dynamics over a longer period we next increased the imaging interval, which reduces photobleaching and potential phototoxic effects – images were captured once a minute for 1 h. This regime revealed the emergence of thin filopodia from the lateral protrusions (Fig. 6A; Movies 20, 21). These lateral filopodia had a homogeneous thickness and were extended on average five times per hour (Fig. 6B,C). Lateral filopodia were longer than the lateral protrusions themselves, and the combined length of protrusions and filopodia averaged 4.7 μm (Fig. 6D); together they can extend within the neuroepithelium beyond their immediate cell neighbours. Taken together, these findings show that both actin and microtubules extend within the lateral protrusions, that these structures have lamellipodia-like characteristics and that emerging filipodia extend the reach of lateral protrusions.

### Developing neuroepithelial cells do not share mitochondria via lateral protrusions

To investigate whether lateral protrusions act as tunnelling nanotubes within the neuroepithelium, we misexpressed the mitochondrial marker mNeonGreen–TOMM20 and the membrane marker pm-mKate2. We then employed a fast imaging protocol to track both mitochondrial movements and cell membrane dynamics. Despite the dynamic nature of mitochondria within the cell cytoplasm, transfer between cells was never observed (Fig. S4A,B, Movie 22). We further generated and analysed electron microscopy images of the apical neuroepithelium of developing chick and human spinal cord for mitochondria localisation. These organelles were found in the apical endfoot in both species but were not located in lateral protrusions and appeared to be too large to enter these structures (Fig. S4C,D). These live-imaging and EM data, together with the finding that lateral protrusions from non-adjacent cells extend along each other (Fig. 4D,E) suggest that the meeting of lateral protrusions does not lead to formation of cytoplasmic connections between cells.

### Lateral protrusions depend on actin but not microtubule dynamics, and lateral filipodia are also actin dependent

**Fig. 7.**
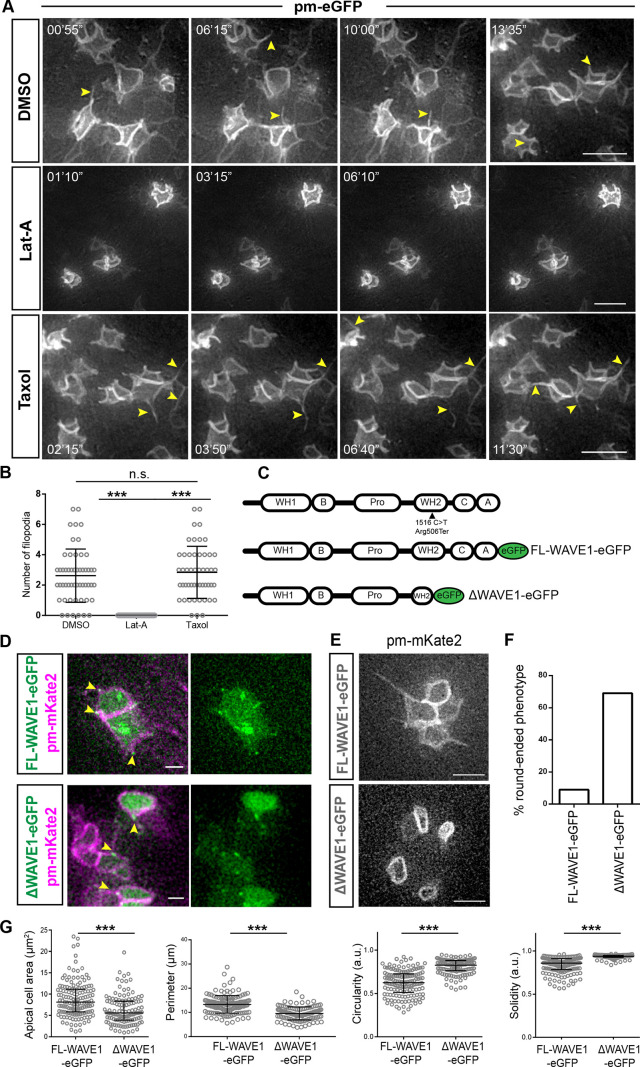
**Lateral protrusion dependence on actin and WAVE1, and ΔWAVE1 round-ended apical endfoot phenotype.** (A) Live-imaging timepoints of cells expressing the membrane marker pm-eGFP, monitoring lateral protrusions and filopodia in the presence of latrunculin A (Lat-A), Taxol or DMSO vehicle control. Arrowheads, filopodia. (B) Quantification of filopodial protrusions in each condition in A. Latrunculin A treatment inhibits filapodial extensions compared to DMSO control and Taxol treatments. Error bars show the mean±s.d. DMSO: three experiments, six explants, 44 cells; latrunculin A: three experiments, nine explants, 337 cells; Taxol: two experiments, five explants, 50 cells. ***P<0.0001; n.s., not significant, *P*=0.3852 (one-way ANOVA with Tukey's multiple comparisons test). (C) Schematic of domain composition of the human WAVE1 protein and the nonsense mutation (Arg506Ter) identified by Ito et al. (2018), and details of constructs used for experiments: full length (FL-WAVE1) and truncated protein (ΔWAVE1) with C-terminal eGFP tag. A, acidic domain; B, basic region; C, central domain; Pro, proline-rich domain; WH, WASP homology domain. (D) Upon low-level misexpression, FL-WAVE1–eGFP and ΔWAVE1–eGFP both localised in the cell membrane at the base of lateral protrusions, as shown in representative images of cell co-expressing pm-mKate2 (indicated with arrowheads). (E) Round-ended apical endfoot phenotype in cells misexpressing ΔWAVE1–eGFP, but not FL-WAVE1–eGFP. Images extracted from Movies 26 and 27. (F) Percentage of cells with round-ended phenotype following FL-WAVE1–eGFP and ΔWAVE1–eGFP misexpression. FL-WAVE1–eGFP: 12 of 133 cells (9%) from four experiments, eight slices. ΔWAVE1–eGFP: 116 of 167 cells (69%) from two experiments, four slices. (G) Quantification of apical endfoot shape (using cell area, perimeter, circularity and solidity parameters; a.u., arbitrary units) in cells expressing ΔWAVE1–eGFP or FL-WAVE1–eGFP. Note, classically defined ‘roundness’ remained unchanged (*P*=0.2288). FL-WAVE1–eGFP: two experiments, four explants, 120 cells. ΔWAVE1–eGFP: four experiments, nine explants, 146 cells). Error bars indicate median with interquartile range. ***P<0.0001 (unpaired two-tailed *t*-test). Scale bars: 10 μm (A), 5 μm (E), 2 μm (D).

To address whether actin or microtubule dynamics are necessary for the formation of lateral protrusions, the membrane marker pm-eGFP was introduced into the neuroepithelium (as above) and explants were incubated in media containing latrunculin A (1 μM), Taxol (10 μM) or control vehicle DMSO for 1 h, followed by live imaging. Latrunculin A binds to free actin dimers and depletes the pool of available actin for filament formation and maintenance, resulting in actin filament depolymerisation over time (Coué et al., 1987). Taxol binds bundles and stabilises microtubules, perturbing depolymerisation events (Schiff and Horwitz, 1980). Exposure to latrunculin A reduced lateral protrusion dynamics and led to quantifiable inhibition of lateral filopodia formation, whereas Taxol had no effect on these structures (Fig. 7A,B; Movies 23–25). These data show that lateral protrusions and filopodia rely on actin but not microtubule dynamics for their extension.

### Lateral protrusions rely on the actin-binding protein WAVE1

To monitor and manipulate actin dynamics on a cell-by-cell basis and more specifically in lateral protrusions, we looked for an actin regulatory protein that might act in these structures. Branched actin filament networks are assembled through the combined activities of the Arp2/3 complex and different forms of WASP/WAVE family proteins. In *Drosophila*, loss of SCAR/WAVE1 complex activity in notum epithelium results in specific loss of basolateral protrusions (Georgiou and Baum, 2010). TIRF imaging has also shown that in comparison with WAVE2 (also known as WASF2) and N-WASP (also known as WASL), WAVE1 generates shorter, slower growing actin filaments (Miki et al., 1998; Sweeney et al., 2015; Yamazaki et al., 2005), consistent with the form and dynamics of lateral protrusions we observe here. Moreover, mutations in human WAVE1 Arp2/3 and actin-binding domain lead to inhibition of fibroblast lamellipodial protrusions (Ito et al., 2018), further identifying WAVE1 as a potential mediator of lateral protrusions. As this region is highly conserved across species (Ito et al., 2018), we cloned and eGFP-tagged the full-length (FL) human WAVE1 and a C-terminal truncated form of the protein (informed by the nonsense mutation described by Ito et al.; Fig. 7C). This truncation generates a protein lacking the C and A domains and part of the WH2 domain necessary for binding actin and the Arp2/3 complex to promote actin polymerisation (Boczkowska et al., 2014; Ito et al., 2018). We predicted that misexpression of truncated (Δ) WAVE1–eGFP would have a dominant-negative action, competing with endogenous WAVE complex-binding partners.

When misexpressed at low levels, FL-WAVE1–eGFP or ΔWAVE1–eGFP localised at cell–cell junctions and at the base of lateral protrusions (Fig. 7D). When ΔWAVE1–eGFP was expressed, most lateral protrusions were lost (some transient filopodia were observed; Movie 27) and the endfoot acquired a round-ended appearance. In contrast, this phenotype was only occasionally observed following high level WAVE1–eGFP misexpression (Fig. 7E,F; Movies 26, 27; see Materials and Methods for phenotype criteria). Moreover, quantification of apical endfoot shape revealed that cells expressing ΔWAVE1–eGFP had a smaller apical endfoot area and shorter perimeter while possessing greater circularity and solidity than cells expressing FL-WAVE1–eGFP (Fig. 7G). These data show that apical endfoot shape and lateral protrusion dynamics rely on WAVE1; furthermore, they indicate that misexpression of ΔWAVE1 can be used to interfere with lateral protrusion formation on a cell-by-cell basis.

### Loss of lateral protrusions neither alters expression of adherens junction protein N-cadherin nor disrupts neuroepithelial tissue integrity

**Fig. 8.**
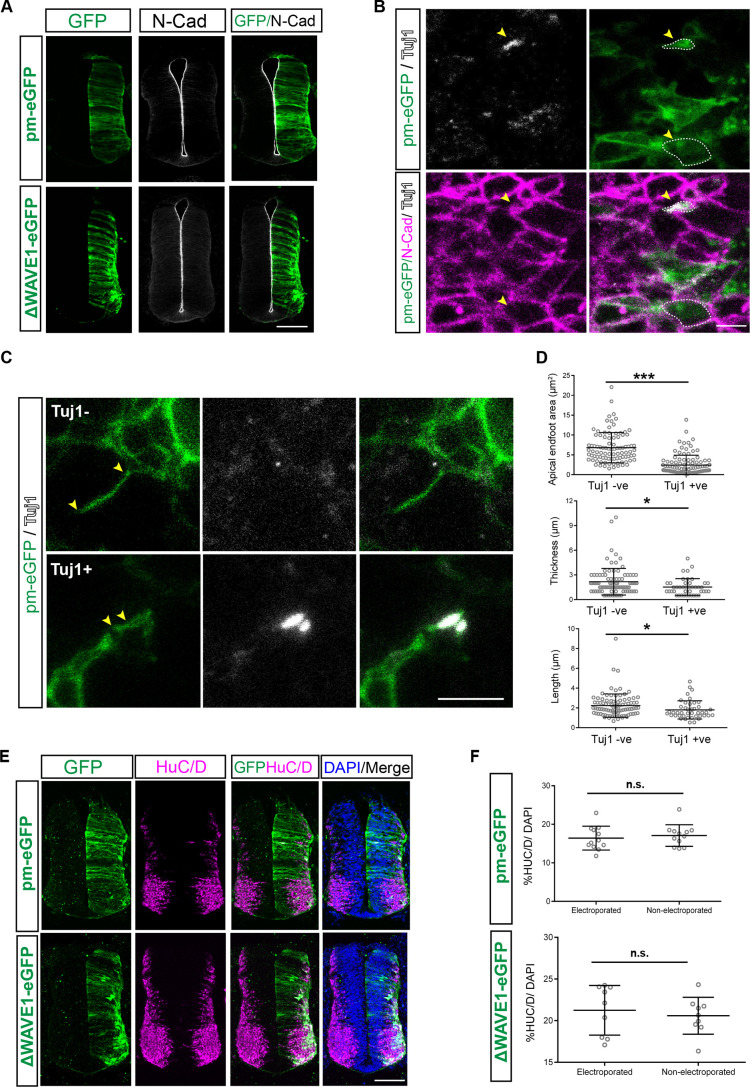
**ΔWAVE1 misexpression does not alter N-cadherin expression, and although neurogenesis involves lateral protrusion withdrawal, ΔWAVE1 does not trigger neurogenesis.** (A) No alteration in the expression of adherens junction protein N-cadherin following misexpression of control pm-eGFP or ΔWAVE1–eGFP in neural tube for 48 h. Images are representative of >3 transverse sections from each of four embryos for pm-eGFP and ΔWAVE1–eGFP. Embryos are shown with electroporated sides on the right and non-electroporated sides on the left. (B) TuJ1^+^ and TuJ1^−^ apical endfeet of pm-eGFP-expressing cells viewed en face at the level of adherens junctions (marked by N-cadherin expression, pink). The upper arrowhead indicates a TuJ1^+^ cell, while the lower arrowhead indicates a TuJ1^−^ cell. White dashed lines show examples of areas measured. (C) Representative images showing lateral protrusion length measurement (between yellow arrowheads) in TuJ1^+^ and TuJ1^−^ cells (see Movies 28, 29). (D) Dot plots showing significant difference in apical endfoot area, and lateral protrusion thickness and length between TuJ1^−^ and TuJ1^+^ cells. Apical endfoot area: four experiments, 10 explants, 103 TuJ1^+^ cells, 93 TuJ1^−^ cells; ***P=0.0001. Lateral protrusion thickness and length: four experiments, 43 TuJ1^+^ cells, 93 Tuj1^−^ cells; thickness, *P=0.0172; length, *P=0.0335 (two-tailed unpaired *t*-test). (E) No alteration in numbers of neurons (HuC/D^+^ cells) following misexpression of control pm-eGFP or ΔWAVE1–eGFP in the neural tube for 48 h. Embryos are shown with electroporated sides on the right and non-electroporated sides on the left. Embryos were stained for HuC/D, and nuclei are labelled with DAPI. (F) Percentage of HuC/D^+^ cells on electroporated and non-electroporated sides of the neural tube (>3 transverse sections from each of three embryos for pm-eGFP and four embryos for ΔWAVE1–eGFP). n.s., not significant; pm-eGFP, *P*=0.7496; ΔWAVE1–eGFP, *P*=0.4266 (two-tailed unpaired *t*-test). Error bars in D and F show mean±s.d. See Fig. S6 and note that the apparent increased basal localisation of cell bodies in E following ΔWAVE1–eGFP misexpression may reflect the cytoplasmic localisation of this protein in comparison with membrane localisation of pm-GFP. Scale bars: 100 μm (A,E), 5 μm (B,C).

To address the functional significance of lateral protrusions, we first investigated whether their loss affected maintenance of adjacent adherens junctions. Localisation of the adherens junction constituent protein N-cadherin appeared unaltered at the apical surface across the dorsoventral axis of the neural tube following ΔWAVE1–eGFP misexpression, suggesting that adherens junctions remain intact despite the loss of lateral protrusions (Fig. 8A). The developing, neuron-generating neuroepithelium lacks tight junctions, which are initially located sub-apically, below the adherens junctions (Aaku-Saraste et al., 1996). This raises the possibility that sub-apical protrusions facilitate neuroepithelial integrity. To test this, Texas Red-labelled 70 kDa dextran was injected into the neural tube lumen, and dextran leakage into the neuroepithelium in the presence of ΔWAVE1–eGFP or control pm-eGFP was assessed; however, no differences were apparent after 24 h (Fig. S5). These data indicate that within this developmental window, loss of lateral protrusions neither alters adherens junctions between cells nor impacts neuroepithelial tissue integrity.

### Neuronal differentiation involves withdrawal of lateral protrusions, but precocious loss of these protrusions is insufficient to trigger neurogenesis

Neuronal differentiation involves the delamination of newborn neurons, during which they are released from apical connection with neighbouring cells (Itoh et al., 2013; Kasioulis and Storey, 2018; Rousso et al., 2012; Zhang et al., 2010). This includes an abscission process that operates at the level of the adherens junctions (Das and Storey, 2014) and may also involve withdrawal of the lateral protrusions described here. To investigate whether this latter step is involved in the regulation of neurogenesis, we first compared protrusion characteristics in TuJ1 (TUBB3)-expressing newborn neurons (which have yet to delaminate) and neural progenitors. To increase the number of newborn neurons, we drove this process by misexpressing the proneural factor neurogenin2 (Neurog2–IRES–GFP) together with pm-eGFP in the neural tube (as above). Protrusion dimensions were measured in fixed tissue en face following immunofluorescent detection of TuJ1 and of N-cadherin as a marker of adherens junctions. TuJ1-expressing newborn neurons had a smaller apical endfoot area (measured at the level of the adherens junction) and exhibited significantly reduced lateral protrusion thickness and length (Fig. 8B–D; Movies 28, 29). These data suggest that differentiating neurons normally withdraw their lateral protrusions prior to delamination.

This finding raised the possibility that ΔWAVE1–eGFP-expressing cells lacking lateral protrusions might undergo precocious neuronal differentiation. To test this, ΔWAVE1–eGFP or control pm-eGFP was misexpressed in the neural tube and assessed after 48 h. Cells expressing the neuronal marker HuC/D were then quantified; however, the proportion of HuC/D-expressing cells was unaltered between electroporated and non-electroporated sides in ΔWAVE1–eGFP-expressing and control embryos (Fig. 8E,F). We further analysed this result by comparing the percentage of eGFP^+^ HuC/D^+^ and eGFP^−^ HuC/D^+^ cells within individual embryos in both ΔWAVE1–eGFP and control pm-eGFP conditions using two-tailed paired *t*-tests. This revealed a significant difference between eGFP-expressing and non-eGFP-expressing cells for both experimental and control conditions, while a two-tailed unpaired *t*-test showed that there was no significant difference in the percentage of eGFP^+^ HuC/D^+^ cells between these two conditions (Fig. S6A–C). This indicates that a subset of cells is transfected in both these contexts and further confirms that introduction of the ΔWAVE1–eGFP does not affect neuronal differentiation any more than the control construct. To control for the potential effect of electroporation, we also compared the percentage of cells expressing HuC/D on electroporated and non-electroporated sides within each section for the two conditions, and this revealed no statistical difference (Fig. S6D,E). Taken together, these findings suggest that although withdrawal of lateral protrusions is regulated by the neuronal differentiation programme, this step alone is insufficient to trigger this process.

## DISCUSSION

This study provides a comprehensive live view of cellular protrusions that extend from the apical neuroepithelium and uncovers a sub-apical latticework of lateral protrusions and associated fine filopodia that mediate membrane contacts between non-neighbouring cells within this tissue. The presence of growing microtubules as well as actin in lateral protrusions, the localised expression of ENA and VASP proteins and lack of atypical myosin X in these structures, together with their lamellipodia-like morphology, distinguished these structures from cytonemes. The absence of mitochondria, along with the overlap rather than fusion between lateral protrusions from distant cells, further discriminate these structures from tunnelling nanotubes. Despite the presence of microtubules, lateral protrusions and their extending filopodia were only dependent on actin polymerisation. We further show that the actin-nucleating protein WAVE1 localises to lateral protrusions and that introduction of mutant WAVE1 leads to their loss. Importantly, this did not alter localisation of the adherens junction protein N-cadherin, nor did it disrupt neuroepithelial tissue integrity. While lateral protrusions were specifically withdrawn prior to delamination of newborn neurons, experimentally induced precocious loss of these structures was insufficient to trigger neurogenesis. These findings suggest that lateral protrusion withdrawal is regulated by the neuronal differentiation programme and that reconfiguration of the actin–WAVE1 network is an early step that anticipates neuronal delamination. Overall, this study identifies a dynamic sub-apical latticework of lateral protrusions that has the potential to mediate cell–cell communication across the developing neuroepithelium.

Ultrastructure analyses and live imaging of the lumen-facing apical membrane of neuroepithelial cells revealed three distinct apically protrusive structures, in addition to the primary cilium; microvilli (also known as microspikes), lamellopodia-like structures and long filopodia, all of which could be further visualised with F-tractin and so are actin-based structures. The presence of microspikes on neuroepithelial cells has been reported previously using electron microscopy to analyse the closing neural tube in chicken and rodent embryos (Shepard et al., 1997, 1993; Smith et al., 1982; Waterman, 1976). Later work revealed localisation of the surface glycoprotein prominin-1 (CD133) on neuroepithelial cell microvilli (Marzesco et al., 2005), suggesting that these serve to increase the apical surface area for internalisation of potential growth factors present in the lumen. Most recently, in Caco-2 intestinal epithelial cells, actin assembly has been shown to drive microspike protrusions toward apical adherens junctions and increase the surface membrane available for cadherin-mediated cell–cell adhesion (Li et al., 2021, 2020), which might be conserved in the neuroepithelium. We further document, to our knowledge, novel apical lamellipodial protrusions, the dimensions of which suggest that they can reach over neighbouring apical endfeet and so may influence their neighbours and/or operate to sense the immediate environment. Finally, we observed long apical filopodia that contact non-neighbouring cells. Such protrusions have been noted previously in fixed tissue (Shepard et al., 1997, 1993; Waterman, 1976). We reveal here that these apical filopodia exhibit retrograde membrane movement indicative of active endocytosis of components from the environment. In support of this possibility, signalling pathways such as sonic hedgehog and Wnt show the binding and trafficking of ligands along such cytoneme-like membrane protrusions in the chick limb mesenchyme (Sanders et al., 2013) and the early zebrafish neural plate (Stanganello et al., 2015).

The presence of lateral protrusive structures has been reported previously in a range of non-neural epithelia (Demontis and Dahmann, 2007; Farquhar and Palade, 1965; Tang and Brieher, 2012). Recent work in developing mouse cerebral cortex has identified very similar lateral lamellipodia-like structures by electron microscopy and reported their actin dependence (Shinoda et al., 2018). We confirm the sub-apical emergence of lateral protrusions in the chicken and human spinal cord using TEM. In the chick, live imaging further revealed that these lamellipodia-like protrusions create contacts between non-neighbouring cells and provide a platform for filopodial extensions, forming dynamic membrane contacts with a radius of at least two apical endfeet (greater than ∼5 μm) from each neuroepithelial cell – effectively forming a latticework of cell contacts within the neuroepithelium. Our findings suggest that these contacts do not involve cell fusion (they extend along each other and appear not to traffic mitochondria) and so are distinct from tunnelling nanotubes (Korenkova et al., 2020; Rustom et al., 2004). However, future studies could assess further potential trafficked organelles, including lysosomes and endosomes.

The lamellipodial-like structure of lateral protrusions shares some similarity with basal protrusions described in *Drosophila* notum epithelium (Georgiou and Baum, 2010). Importantly, these basal protrusions are uniquely SCAR/WAVE dependent, and consistent with this, we found the related branched actin-nucleating protein WAVE1 (Carlier et al., 2015; Pollard and Borisy, 2003) was localised to and required for lateral protrusions. Moreover, WAVE regulatory complexes work cooperatively with Ena/VASP proteins to enhance Arp2/3-mediated actin assembly in lamellipodia (Chen et al., 2014; Damiano-Guercio et al., 2020), and ENA and VASP localisation to lateral protrusion tips here further identifies these as lamellipodia-like structures. However, it is important to note that WAVE1 also localises to mitochondria in fibroblasts (Ito et al., 2018), and we observe this in neuroepithelial cells too, so its regulation of lateral protrusions could also reflect an indirect WAVE1 function in this organelle. Lateral protrusions were additionally distinguished by the presence of polymerising microtubules from base to tip, while most cellular protrusions are exclusively composed of actin or have microtubules confined to the base (González-Méndez et al., 2019). Indeed, together these findings identify a surprising similarity between sub-apical lateral protrusions and their extending filopodia and the cytoskeletal organisation of later forming axonal growth cones (Robert Manak, 2018).

The discovery that lateral protrusions are withdrawn by newborn neurons that are still attached at the apical surface suggests that this step is an early event directed by the neuronal differentiation programme. Moreover, precocious reduction of lateral protrusions induced by misexpression of a mutant form of WAVE1 was insufficient to trigger neurogenesis. Importantly, neuronal delamination and differentiation is elicited by loss of adherens junction constituent proteins N- or E-cadherin (Hatakeyama et al., 2014; Itoh et al., 2013; Rousso et al., 2012). It is therefore not surprising that reduction of WAVE1 and lateral protrusive activity also did not impact N-cadherin expression or localisation. This is further consistent with findings in the *Drosophila* notum epithelium, where loss of SCAR/WAVE1 complex activity similarly does not impact apico-basal polarity (Georgiou and Baum, 2010). These findings suggest that regulation of WAVE1 activity in lateral protrusions is an early action of the neuronal delamination mechanism and supports the notion that this anticipates the extensive actin network reconfiguration apparent during apical abscission (Das and Storey, 2014; Kasioulis et al., 2017). The importance of actin configuration in this process is further underlined by the requirement for the actin crosslinking protein filamin A, mutation of which underlies defective neuronal delamination in the human condition periventricular heterotopia (Parrini et al., 2016; Sheen et al., 2001; Zhang et al., 2013).

Overall, our findings indicate that withdrawal of lateral protrusions is regulated by, but does not drive, the neuronal differentiation programme and that these structures may be part of the mechanism that apically anchors cells within the neuroepithelium. However, this does not exclude roles in cell communication for this latticework of cell membrane contacts (Fig. S7). One possibility is that lateral protrusions (which with their filopodia radially contact cells several endfeet away) facilitate cell–cell coupling within cell micro-clusters, which have recently been shown to exhibit synchronous Hes gene (Notch signalling) oscillations, and so may serve to regulate neuron production (Biga, 2021). Indeed, a role in local coordination of cell signalling is consistent with lateral protrusion withdrawal in newborn neurons, which might then experience reduced signalling from neighbouring cells, while subsequently delivering Notch ligand via adherens junctions (Hatakeyama et al., 2014; Falo-Sanjuan and Bray, 2021). This an interesting idea, because it could provide a cell biological basis for the compartmentalisation of cell selection within the developing neuroepithelium. The lateral protrusion latticework also provides an opportunity to relay signals across the developing neuroepithelium. Such relays may be required to convey signalling beyond the reach of ligand diffusion in the extracellular space or neural tube lumen and/or delivery by cytonemes or long filopodia. The potential for lateral protrusions to facilitate comparison and integration of signalling between cells in this further context suggests a role in establishing and/or maintaining positional information across the neuroepithelium. To elucidate these functional roles, future studies should assess local patterns of contact dynamics and localise ligands and receptors in lateral protrusions.

## MATERIALS AND METHODS

### Tissue sources

Fertilised Bovans Brown chicken eggs were supplied by Henry Stewart & Co. Ltd and incubated at 38°C to the desired Hamburger and Hamilton (HH) stage. Work with chicken embryos at the stages investigated here do not require ethical approval. Fixed human embryonic spinal cord tissue at Carnegie stage (CS)12 used for TEM studies was provided by the Human Developmental Biology Resource (HDBR; www.hdbr.org), under registered project # 200407. The HDBR obtains appropriate maternal written consent and approval from the London Fulham Research Ethics Committee (18/LO/0822) and the Newcastle and North Tyneside NHS Health Authority Joint Ethics Committee (08/H0906/21+5). HDBR is regulated by the UK Human Tissue Authority (HTA; www.hta.gov.uk) and operates in accordance with the relevant HTA codes of practice and according to the principles expressed in the Declaration of Helsinki.

### Cloning

The ImProm-II reverse transcription system (Promega, A3800) was used to generate cDNA from human embryonic stem cell (hESC; H9) RNA. Full-length WAVE1 was PCR amplified, adding in a linker sequence. The WAVE1 PCR product was then used to amplify the mutant WAVE1 (ΔWAVE1; a truncated version of the gene lacking sequence distal to nonsense mutation Arg506Ter; Ito et al., 2018; see Fig. 7C) to which the linker sequence was added in a second round of PCR. Both were tagged with monomeric eGFP, separated by the linker sequence, in a further PCR. Primers for full-length WAVE1 (FL) and ΔWAVE1 are provided in Table S1. PCR reactions were carried out using the Phusion polymerase (Thermo Fisher Scientific, F537). Following each reaction, the products were gel extracted using the Zymoclean Gel DNA recovery kit (D4001). The pENTR/D-TOPO cloning kit (Thermo Fisher Scientific, K240020) was used to introduce the PCR products into the entry vector for Gateway cloning. PCR products in the entry vector were then recombined in the destination vector using the LR clonase II enzyme mix (Thermo Fisher Scientific, 11791100). We utilised the PBX destination vector (kind gift from Timothy Sanders, The Neuroscience Institute, University of Chicago, IL, USA; Sanders et al., 2013) which contains the CAG promoter, for efficient expression in chicken tissue.

### *In ovo* electroporation

Fertilised chicken eggs were incubated at 38°C to stage HH10–12, and the neural tube was electroporated with a single construct or combination of constructs at concentrations given in Table S2. We targeted the neural tube region posterior to the forelimb level and along the dorsoventral axis. Transfected cells were typically located throughout the dorsal region and extended to include the Olig2-positive ventral domain. To obtain mosaic expression pattern, neural tubes were electroporated with three 50 ms pulses, separated by a 950 ms gap, at 17 V using the ECM 830 square wave electroporation system (BTX). Details of plasmid constructs, source and dilutions used are provided in Table S2.

### Embryonic tissue explant culture

Electroporated embryos were allowed to develop overnight to stage HH17–18, and the neural tube in the interlimb region was then bisected along the dorsoventral axis to gain access to the neuroepithelial apical surface. The non-electroporated side was discarded, and hemi neural tube explants (∼2–3 somite lengths), including associated somites, were embedded for en face imaging, with the neuroepithelial apical side orientated to face the base of the culture dish. In most experiments only one explant per embryo was used. Explants were embedded in collagen [supplemented with 0.1% acetic acid, 5× L-15 medium (Thermo Fisher Scientific, 41300) and 7.5% sodium bicarbonate (Thermo Fisher Scientific, 25080094)] in poly-D-lysine coated glass-bottomed Petri dishes (World Precision Instruments, FD35-PDL-100) and covered with neurobasal medium (Thermo Fisher Scientific, 12348017) supplemented with B-27 (Thermo Fisher Scientific, 17504044), GlutaMAX (Thermo Fisher Scientific, 35050038) and gentamicin (Thermo Fisher Scientific, 15750037). The plates were then placed in a 37°C-maintained incubator supplied with 5% CO_2_, giving time for the explants to recover for 2 h before commencing live imaging (Das et al., 2012).

### Time-lapse imaging

Time-lapse imaging of neural tube explants was performed using a Deltavision Core microscope system in a WeatherStation environmental chamber maintained at 37°C (GE Healthcare). Imaging was optimised for minimal light transmission, exposure times (50–100 ms) and total acquisition times (10–20 min) to avoid phototoxicity (Das et al., 2012; Das and Storey, 2014). Image acquisition was performed using an Olympus 60×1.42 NA oil immersion objective or an Olympus 40×1.25 NA silicone oil immersion objective, a solid-state LED light source and a CoolSnap HQ2 cooled CCD camera (Photometrics). Unless otherwise stated, 10 optical sections spaced 0.5 μm apart were acquired for each explant (1024×1024 pixels, 1×1 binning). For the live-imaging sessions totalling for 1 h, image acquisition between timepoints was set at 1-min intervals. Images were deconvolved using the SoftWorx image processing software.

### Preparation of tissue for imaging cytoskeletal manipulations

To investigate the effect of actin polymerisation inhibition or microtubule stabilisation, neural tube explants embedded for en face imaging were incubated in pre-warmed neurobasal medium containing latrunculin A (1 μM; Abcam, ab144290) or Taxol (10 μM; Sigma, T7191) for 1 h before imaging.

### Confocal imaging and 3D modelling

Imaging of sections or embedded tissue (Fig. 8A,B,D) was carried out with the Leica SP8 confocal microscope (lenses HC PL APO CS2 20×/0.75 DRY or HC PL APO CS2 63×/1.40 OIL). 3D modelling for Movie 7 was carried out using the IMARIS software (Oxford instruments).

### Immunofluorescence and fixed-tissue imaging

The interlimb region of HH17–18 chick embryos was fixed in 4% paraformaldehyde for 2 h at 4°C, washed with phosphate-buffered saline (PBS) and equilibrated overnight in 30% sucrose at 4°C. The tissue was then embedded in 1.5% LB agar (Sigma, L7025) and 5% sucrose dissolved in PBS. Mounted tissue was dehydrated for 48 h in 30% sucrose and snap frozen on dry ice. Transverse sections, 20–25 μm thick, were then collected using a Leica cryostat (maintained at −25°C). For immunofluorescence experiments, sections were rehydrated in PBS containing 0.1% Triton-X-100 (0.1% Triton-X-100/PBS), incubated in blocking buffer for 1–2 h at room temperature (0.1% Triton-X-100% and 1% heat-inactivated donkey and goat serum, in PBS) and incubated overnight with primary antibodies (diluted in blocking buffer) as in Table S3. Sections were washed with 0.1% Triton-X-100/PBS and incubated overnight with secondary antibodies for 2 h at room temperature. Following washes with 0.1% Triton-X-100/PBS, the sections were mounted in Prolong Gold antifade mountant (Thermo Fisher Scientific, P36930). For en face imaging of fixed tissue, explants were mounted in 0.5% low gelling temperature agarose (Sigma, A9045). For en face confocal imaging following a live-imaging session, the neurobasal medium was immediately replaced with 4% paraformaldehyde for ∼1 h, washed and maintained in PBS. DAPI was added in the PBS washes to stain the nuclei.

### Electron microscopy sample preparation

Chicken embryonic spinal cord at HH12 was fixed in 1% paraformaldehyde and 1.5% glutaraldehyde in 0.05 M cacodylate buffer (pH 7.0) for 2 h at room temperature and was post-fixed with 1% osmium tetroxide (OsO_4_). After washing in cacodylate buffer the tissue was stained with 1% tannic acid in 0.1 M cacodylate buffer for 1 h. The tissue was then dehydrated through an alcohol series finishing in propylene oxide. The tissue was then infiltrated with Durcupan epoxy resin (Sigma), sectioned with a Leica UCT ultramicrotome and imaged on a FEI Tecnai TEM using the SIS Megaview II CCD camera (Fig. 1A–C). Human embryonic tissue fixed in 4% paraformaldehyde and 2.5% glutaraldehyde in 0.1 M sodium cacodylate buffer (pH 7.2) for 60 min was provided by the HDBR. The tissue was washed twice in cacodylate buffer, and small pieces of spinal cord were then post-fixed in 1% OsO_4_ with 1.5% Na ferricyanide in cacodylate buffer for 60 min. After another cacodylate buffer wash they were contrasted with 1% tannic acid and 1% uranyl acetate. The cell pellets were then serially dehydrated into 100% ethanol, changed to propylene oxide, then left overnight in 50% propylene oxide 50% resin and finally embedded in 100% Durcupan resin (Sigma). The resin was polymerised at 60°C for 48 h and sectioned on a Leica UCT ultramicrotome. Sections were contrasted with 3% aqueous uranyl acetate and Reynolds’ lead citrate before imaging on a JEOL 1200EX TEM using a SIS III camera. Chick embryonic tissue imaged in Fig. S4 was prepared using the same protocol as for human tissue.

### Tissue integrity assay using fluorescent dextran

Chick embryos were electroporated with pm-eGFP or ΔWAVE1–eGFP at HH10–12, as above, and at stage HH17–18, the Texas Red-tagged 70 kDa dextran, lysine fixable (Thermo Fisher Scientific, D1864; reconstituted following the manufacturer's instructions) was injected into the neural tube lumen. Embryos were incubated for 1 h and then dissected and fixed immediately with cold 4% paraformaldehyde, followed by gentle washing in PBS to remove the excess dextran, embedding and cryo-sectioning prior to confocal imaging (as above).

### Round-ended phenotype scoring criteria

Cells with a round-ended phenotype were defined as those with apical endfeet with no lateral protrusions and rounded appearance; cells from live-imaging sessions were scored for these features in Fiji (Schindelin et al., 2012) (Fig. 7F). The freehand selection tool was to define apical end foot shape, and Fiji shape descriptors (Analyse, Set measurements, Shape descriptors) were used to derive the measurements presented in Fig. 7G. These measurements of area, perimeter, circularity, aspect ratio (AR), roundness and solidity, were then analysed.

### Software for quantification and analysis

The Softworx software (Applied Precision, Inc), was used for the quantifications in Figs 2B,D, 3A–D,F, 4A, and Fig. S1B. Fiji software (Schindelin et al., 2012) was used for the quantifications in Figs 3F, 6B–D, 7B,F,G, 8D,F, Figs S2A, S3A,C,E. Results of statistical analyses undertaken using Prism 8 are presented in figure legends. Full data and analyses are available on request as metadata files.

## Supplementary Material

Supplementary information

Reviewer comments
