# Supplementary material for: A lateral protrusion latticework connects neuroepithelial cells and is regulated during neurogenesis
Source: J Cell Sci. 2022 Mar 30;135(6):jcs259897. doi: 10.1242/jcs.259897 (PMC8995095; doi:10.1242/jcs.259897)
Supplement: Supplementary information [file joces-135-259897-s1.pdf]

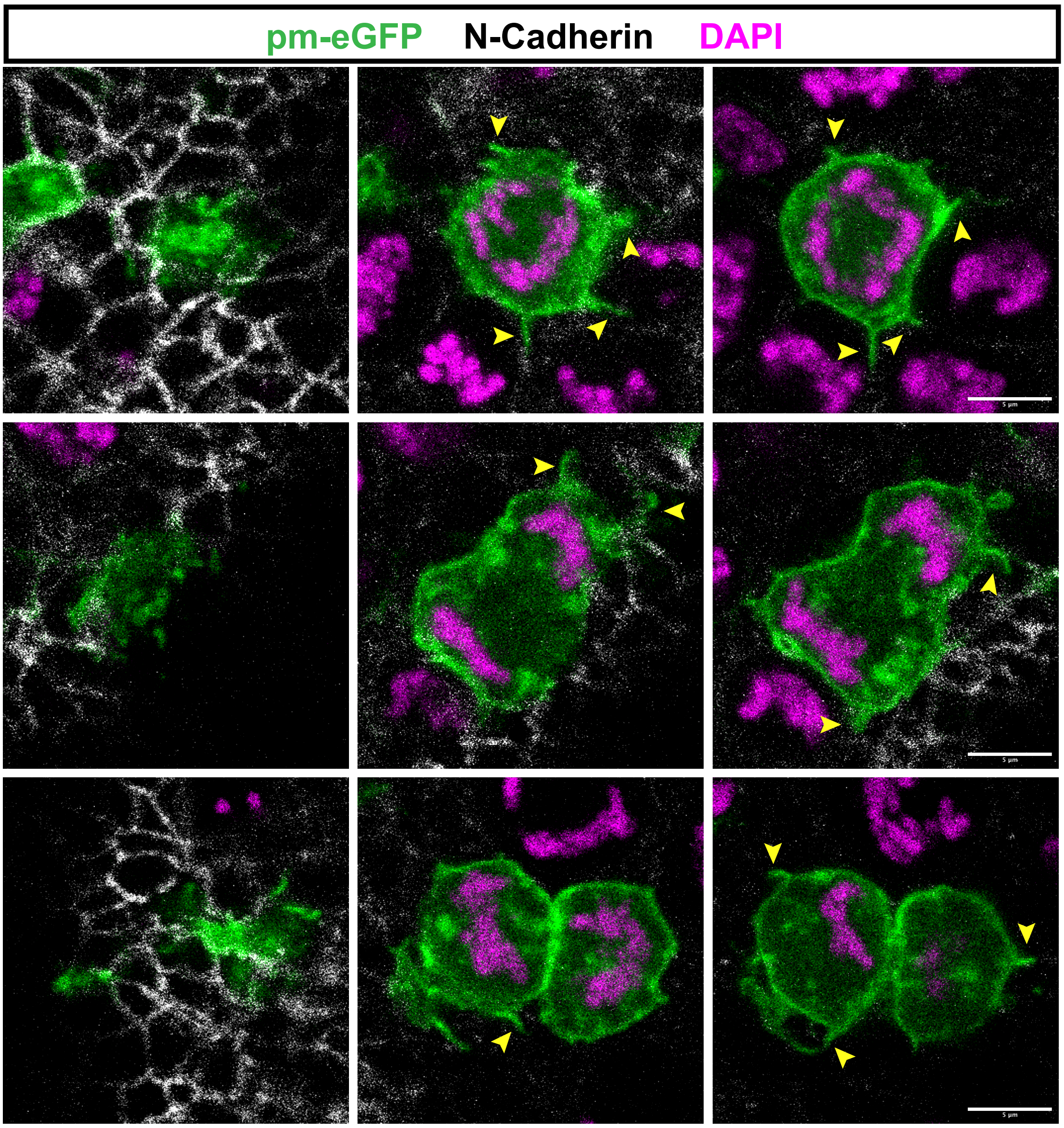

Fig. S1. Lateral protrusions extend from mitotic cells within the neuroepithelium
Three examples of cells undergoing mitosis at the apical surface in fixed tissue, note membrane protrusions (yellow arrows) are present in cells in all phases of this process as revealed by pm-GFP membrane labelling, and chromatin localisation (DAPI). Left hand column low- magnification identification of mitotic cells at the apical surface as indicated by N-Cadherin localisation (representative of 3 experiments, 6 explants, 41 mitotic cells). Scale bars 5 μm.

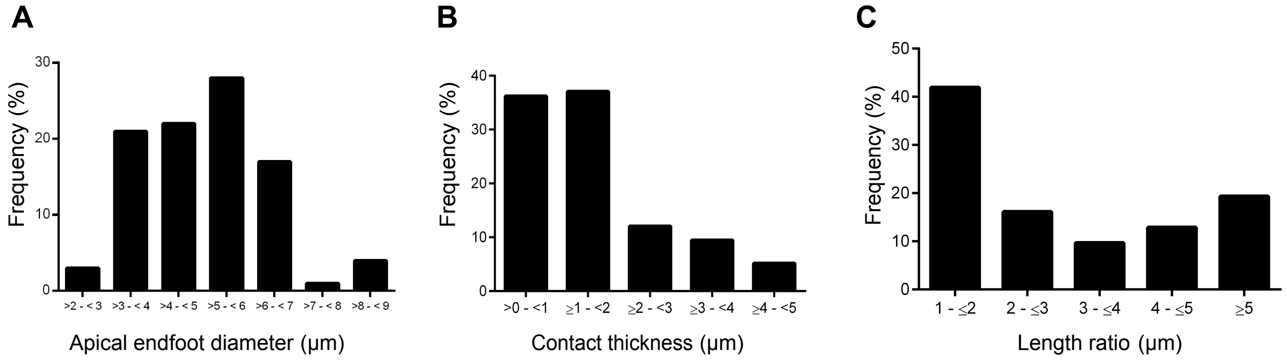

Fig. S2. Apical endfoot and lateral protrusion contact dimensions
(A) Range of apical endfoot diameters, most are between 3 and 6 µm (5 experiments, 9 explants, 98 cells); (B) Contact protrusion thickness between non-neighbouring cells. In more than 70% of protrusion pairs, the contacting thickness did not exceed 2 µm (4 experiments, 7 explants, 116 protrusion pairs); (C) Length ratio between contacting protrusions and their frequency (4 experiments, 8 explants, 31 protrusion pairs).

Journal of Cell Science • Supplementary information

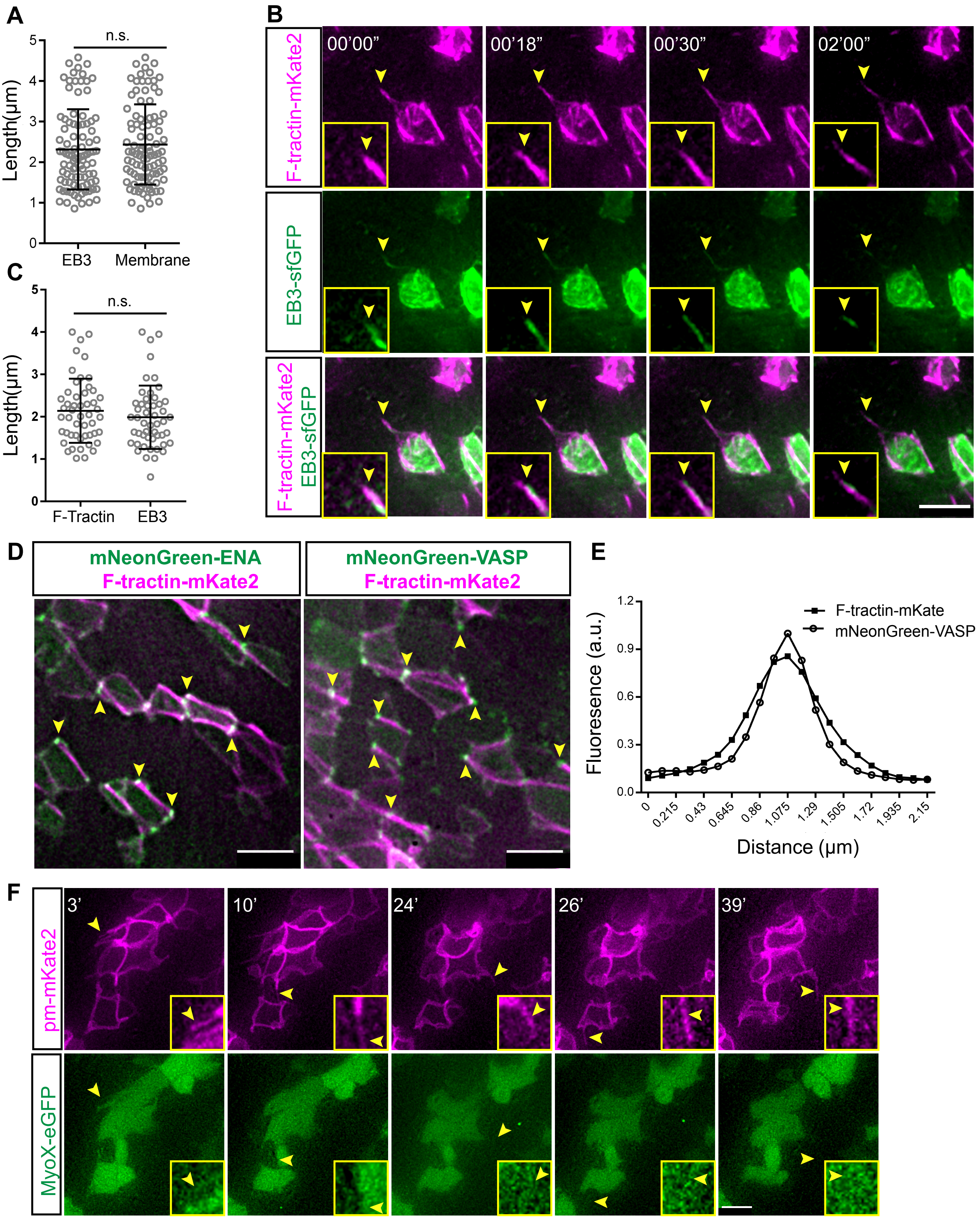

Fig. S3. Lateral protrusions cytoskeletal architecture

(A) Dot plot for maximal length travelled by EB-sfGFP comets in protrusions and the length of lateral protrusions using the membrane marker pm-mKate2 (2 experiments, 5 explants, 93 protrusions; t-test, n.s.= not significant, p = 0.3990); (B) Live imaging timepoints following mis-expression of EB-sfGFP and pm-mKate2, arrowheads and image insets show membrane protrusion tip and EB-sfGFP localization; (C) Dot plot for maximal length travelled by EB-sfGFP comets and the length of F-tractin-mKate2 in the same protrusions (2 experiments, 6 explants, 50 protrusions; t test, n.s. = not significant, p = 0.3104); (D) Live imaging timepoints following mis-expression of mNeonGreen-ENA or mNeonGreen-VASP with F-tractin-mKate2. At the level of adherens junctions, both mNeonGreen-ENA or mNeonGreen localized at cell junctions (yellow arrowheads); (E) Line graph showing fluorescence intensity of mNeonGreen-VASP and F-tractin-mKate2 at the cell junctions (2 experiments, 5 explants, 50 measurements); (F) Live imaging timepoints for MyoX-eGFP and pm-mKate2. MyoX-eGFP was detected in cytoplasm but did not localize to lateral protrusions (arrowheads). (A, C) Mean with standard deviation. Scale bars (B, D, F) 5 μm.

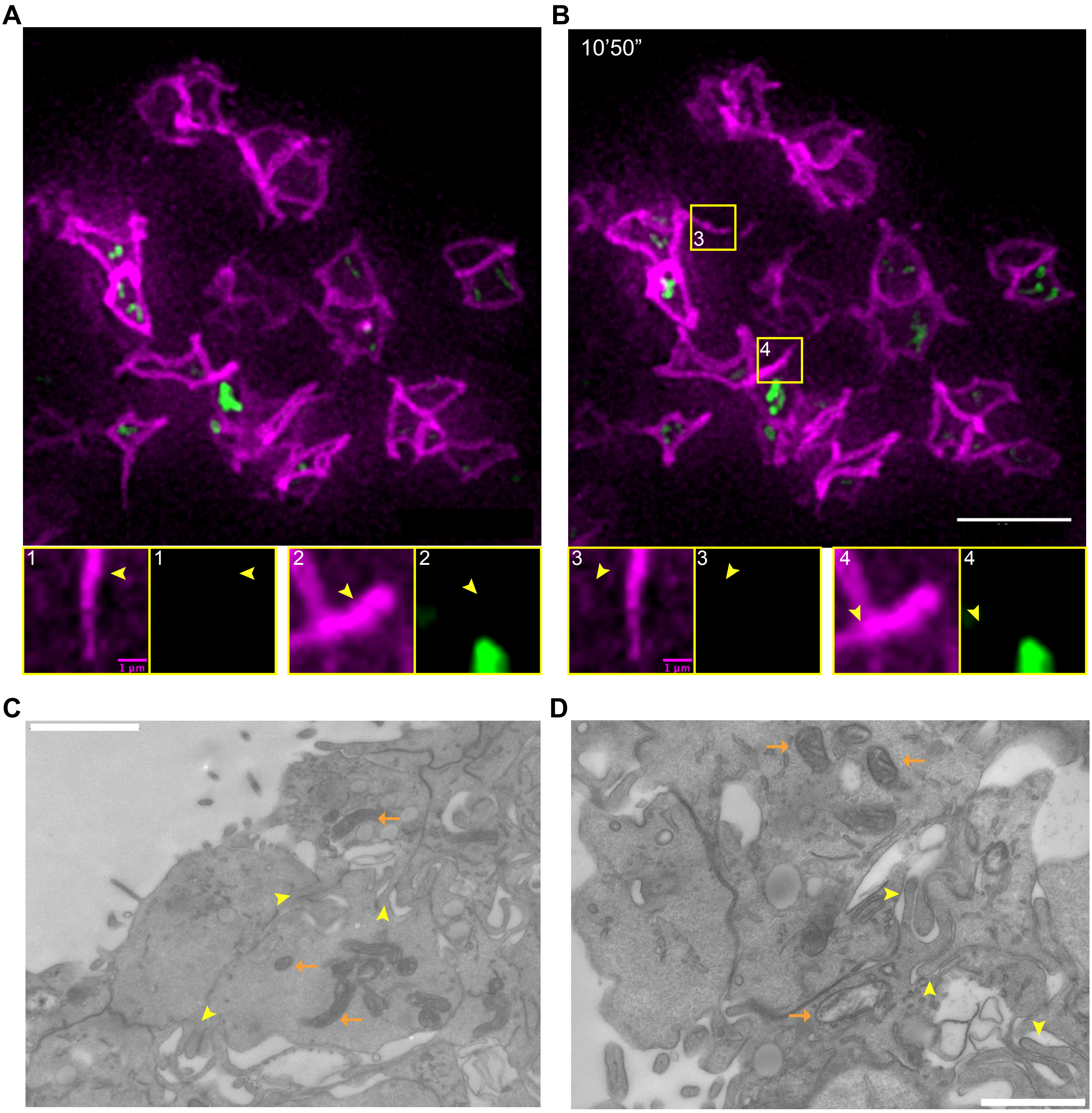

Fig. S4. Mitochondria do not enter lateral protrusions
(A, B) Live imaging timepoints (movie 22) following mis-expression of pm-mKate2 and the mitochondrial marker mNeonGreen-TOMM20. Boxed regions of lateral protrusions are enlarged below, arrowheads indicate lateral protrusions (3 experiments, 9 explants); (C, D) enface EM sections from the apical surface of chick embryonic spinal cord at HH17-18 (sampled in 2 embryos), showing lateral protrusions (yellow arrowheads) and mitochondria (orange arrows). Scale bars (A, B) 10 µm, (C, D) 1 µm.

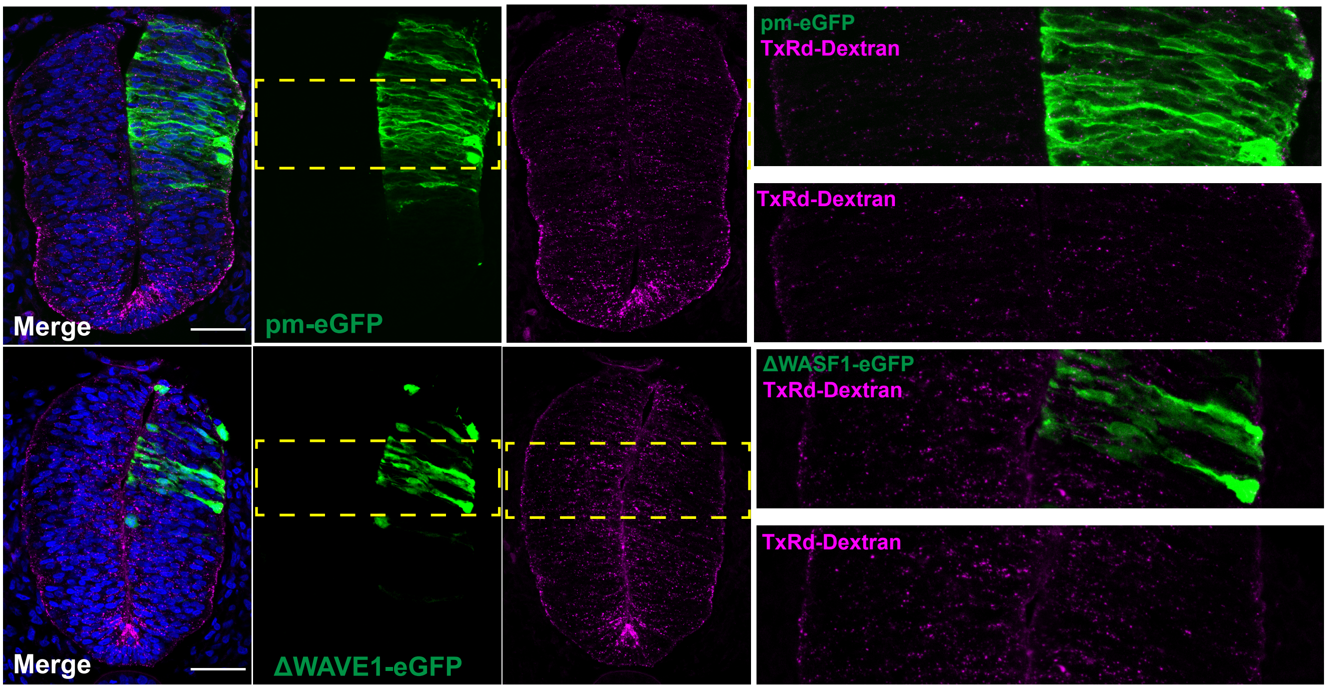

Fig. S5. ΔWAVE1-eGFP does not compromise apical neuroepithelial integrity
Transverse sections through HH17-18 neural tube following misexpression of control pm-eGFP or ΔWAVE1-eGFP and introduction of Texas Red-Dextran (70kDa) into the neural tube for 24h. No qualitative change in the levels of Texas Red-Dextran between the two conditions was observed (pm-eGFP = 4 embryos, ΔWAVE1-eGFP = 4 embryos). Scale bars = 50 μm.

Journal of Cell Science • Supplementary information

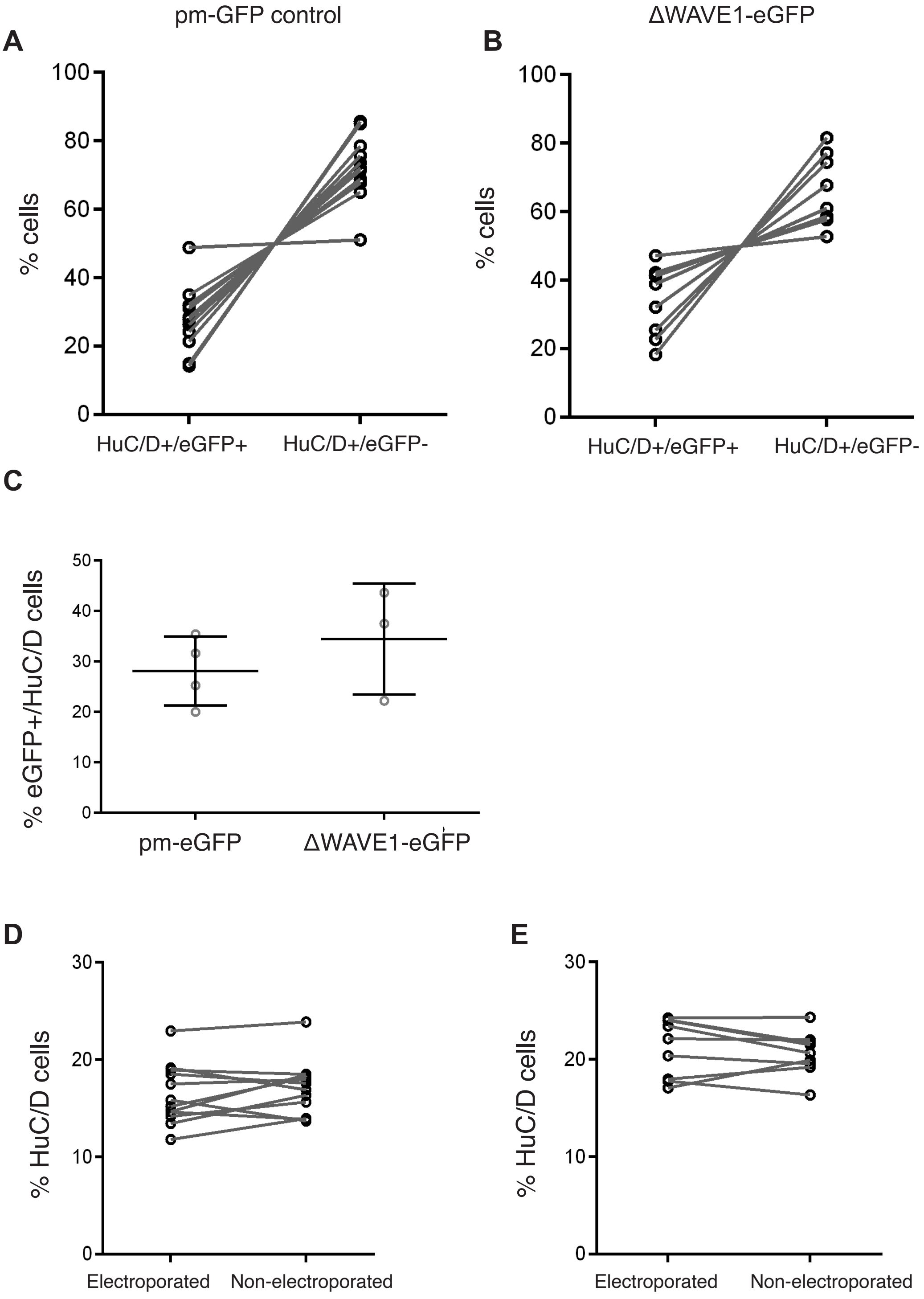

Fig. S6. Analysis of HuC/D/eGFP expressing cells following mis-expression of ΔWAVE1-eGFP
Comparison of percentage of HuC/D+ /eGFP +ve cells and HuC/D+/ eGFP -ve cells within sections from embryos transfected with (A) control pm-eGFP and (B) ΔWAVE1-eGFP using a paired t-test, both comparisons are significantly different between eGFP expressing and non-eGFP expressing cells ($p < 0.0001$ and $p = 0.0017$ respectively); Comparison of the percentage of HuC/D+ /eGFP +ve cells in (C) control pm-eGFP and ΔWAVE1-eGFP conditions, unpaired t-test no significant difference ($p = 0.3838$). Comparison of percentage of cells expressing HuC/D (using DAPI to label and count total cell number) on electroporated and non-electroporated sides within each section for (D) control pm-eGFP and (E) ΔWAVE1-eGFP conditions, compared using a paired t-test, revealed no statistical difference ($p = 0.3281$); Comparison of the percentage of HuC/D+ /eGFP +ve cells in (E) control pm-eGFP and ΔWAVE1-eGFP conditions, unpaired t-test no significant difference ($p = 0.3838$). All comparisons were made using data from (pm-eGFP, 12 sections, 4 embryos, ΔWAVE1-eGFP, 9 sections, 3 embryos), analysis available on request as metadata.

Journal of Cell Science • Supplementary information

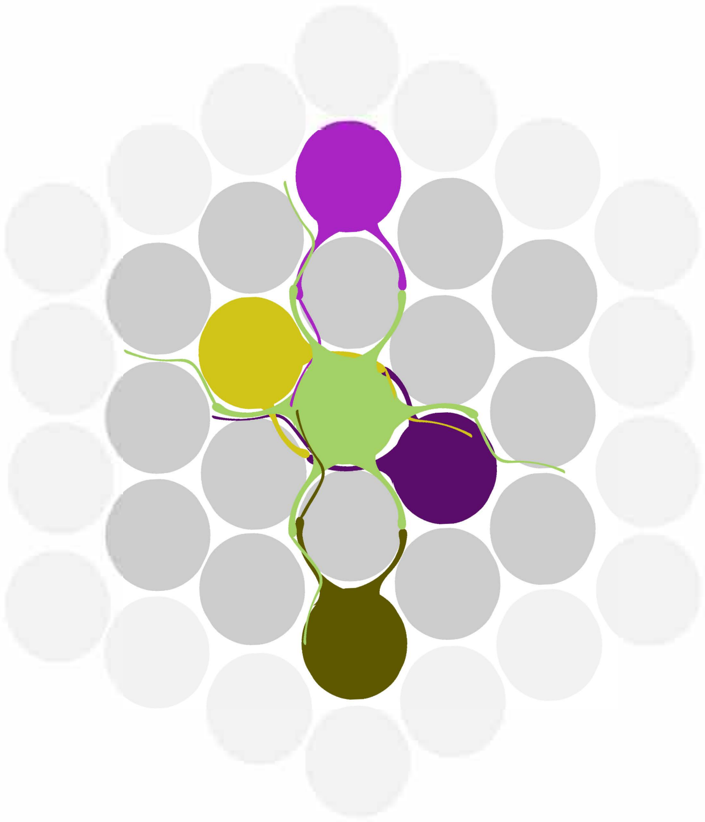

Fig. S7. The lateral protrusion latticework
Schematic depicting exemplar interactions of lateral protrusions extending from one apical end foot (light green). Lateral protrusions with filipodia can make contacts with cells and their protrusions up to two endfeet away (light purple and dark green), while also experiencing mutual contacts with their immediate neighbours (yellow, dark purple). Lateral protrusions from non-neighbouring cells can meet and retract or extend along each other. As all apical endfeet extend lateral protrusions this creates a latticework of short-range cellular protrusions with the potential to mediate cell-cell communication across the neuroepithelium.

Table S1. Primer sequences

Primer	Sequence
WAVE1 Forward	CACCATGCCGCTAGTGAAAAGAAACATCGATCCTAGGCACTTGTGC
WAVE1 Reverse + Linker	GGAACCGCCACCACTCCCGCCACCGGACTCCAACCAATCTACTTCATCAAATTCTGAATCATC
ΔWAVE1 Reverse	TATTGCTTCCAGTAGCACACTCCTGGCATCACTGATTACAG
ΔWAVE1 Reverse + Linker	GGAACCGCCACCACTCCCGCCACCGGATATTGCTTCCAGTAGCACACTCCTGGCATCACTGATTACAG
eGFP Forward	TCCGGTGGCGGGAGTGGTGGCGGTTCCAGCAAGGGCGAGGAGCTGTT
eGFP Reverse	TTCATTACTTGTACAGCTCGTCCATGCC

Table S2. Plasmid constructs, sources, and dilutions

Plasmid	Source	Dilution
pm-eGFP	Tim Sanders, University of Chicago	50 – 100 ng/μl
pm-mKate2	Tim Sanders, University of Chicago	50 – 100 ng/μl
F-tractin-mKate2	Tim Sanders, University of Chicago	50 ng/μl
EB3-sfGFP	Tim Sanders, University of Chicago	25 ng/μl
WAVE1-eGFP	See cloning protocol	1 μg/μl
ΔWAVE1-eGFP	See cloning protocol	1 μg/μl
MyoX-eGFP	Tim Sanders, University of Chicago	1 μg/μl
mNeonGreen-ENA	Allele Biotechnology	1 μg/μl
mNeonGreen-Vasp	Allele Biotechnology	1 μg/μl
mNeonGreen-TOMM20	Allele Biotechnology	1 μg/μl

Journal of Cell Science • Supplementary information

Table S3. Antibodies and stains, sources and dilutions

Antibody	Source	Catalogue number	Dilution
**Primary antibodies**			
Rat N-Cadherin	ThermoFisher	132100	1/300
Tuj1	Biolegend	801213	1/500
Mouse HuC/HuD	ThermoFisher	A21271	1/200
Goat GFP	Abcam	6673	1/500
**Secondary antibodies**			
Donkey anti-mouse 568	ThermoFisher	A-10037	1/300
Donkey anti-rat 568	Abcam	ab175475	1/300
Donkey anti-mouse 647	ThermoFisher	A-31571	1/300
Donkey anti-goat 488	ThermoFisher	A-11055	1/300
Phalloidin CF640R	Biotum	00050	1/300

Journal of Cell Science • Supplementary information

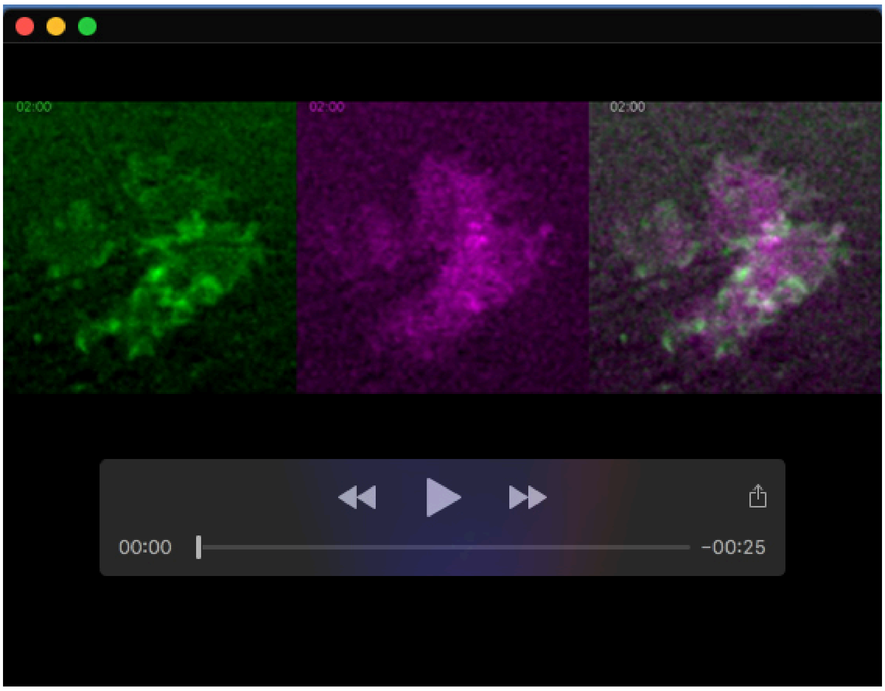

Movie 1. Microvilli formation at the apical cell membrane. These movies are related to Figure 2A. Mis-expressed pm-eGFP labels the plasma membrane and F-tractin-mKate2 the actin cytoskeleton. Maximum intensity projections of deconvolved image sequences were used. Images acquired every 8 seconds.

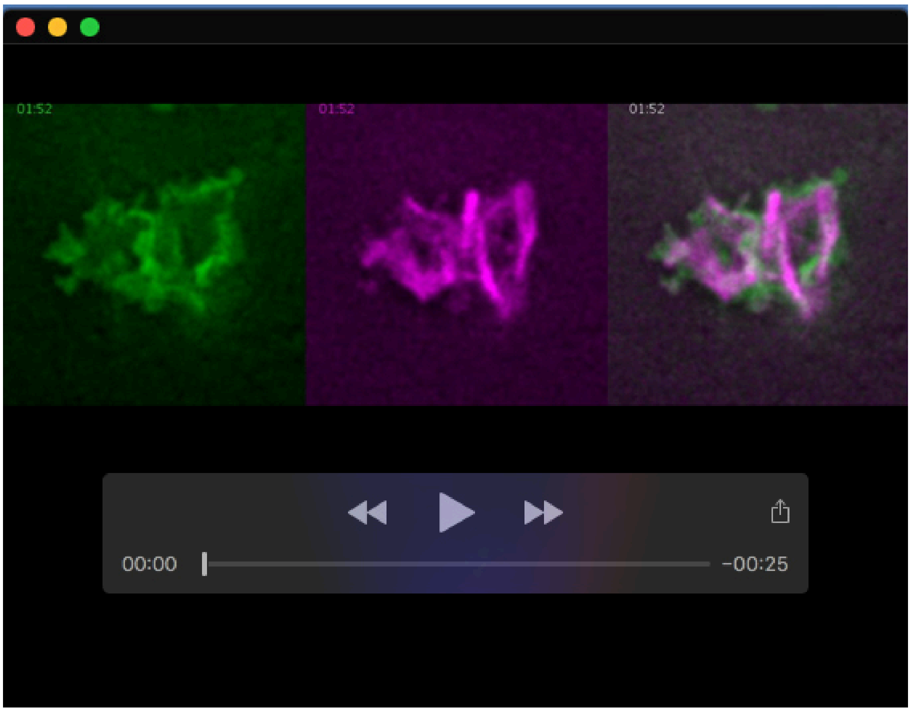

Movie 2. lamellipodial formation at the apical cell membrane. These movies are related to Figure 2A. Mis-expressed pm-eGFP labels the plasma membrane and F-tractin-mKate2 the actin cytoskeleton. Maximum intensity projections of deconvolved image sequences were used. Images acquired every 8 seconds.

Journal of Cell Science • Supplementary information

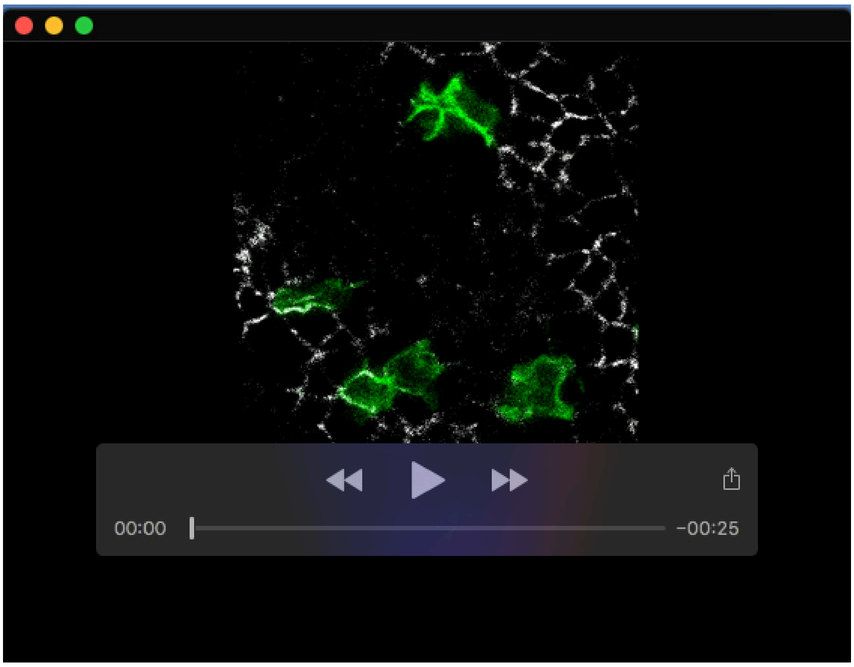

Movie 3. Microvilli formation at the apical cell membrane. This movie is related to Figure 2A. Apico-basal Z-stack series across the apical plasma membrane, labelled with pm-eGFP, sub-apical N-Cadherin based adherens junctions (white) help define apico-basal position.

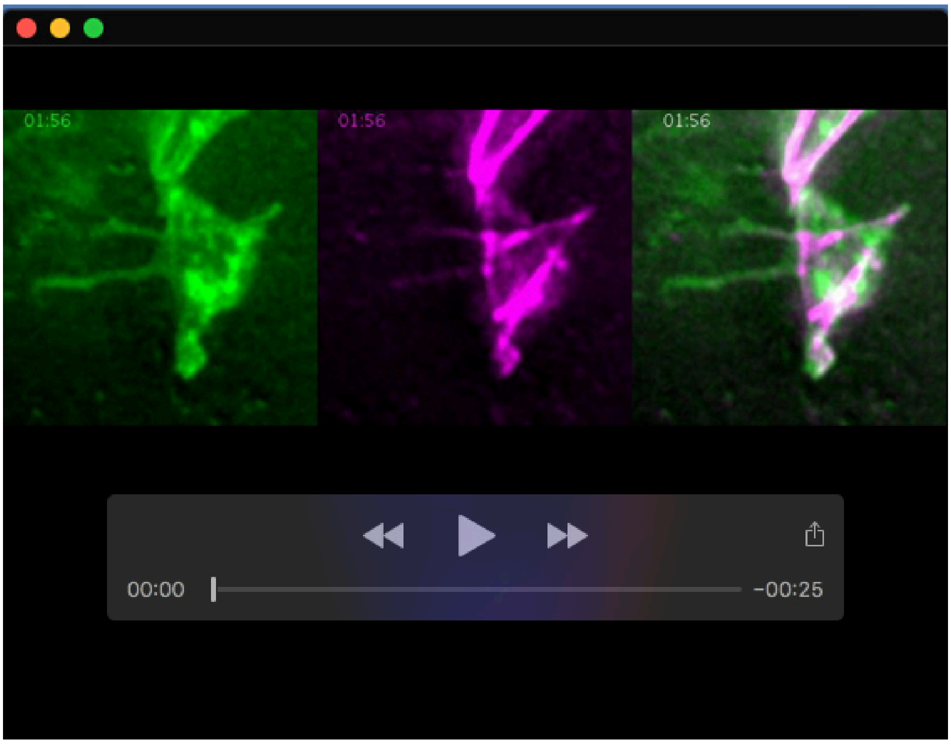

Movie 4. Long thin protrusions extending into the lumen exhibiting retrograde movement of membrane particles. This movie is related to Figure 1C. Mis-expressed pm-eGFP labels the plasma membrane and F-tractin-mKate2 the actin cytoskeleton. Maximum intensity projections of deconvolved image sequences were used. Images acquired every 7 seconds.

Journal of Cell Science • Supplementary information

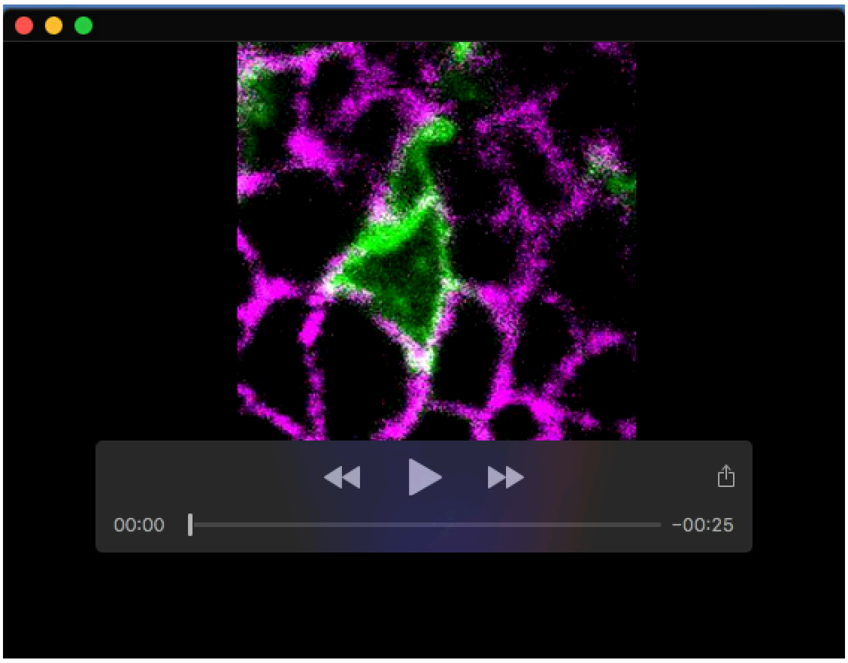

Movie 5. Sub-apical lateral protrusions arise beneath the N-Cadherin based adherens junctions. Apico-basal Z-stack series across the apical plasma membrane, labelled with pm-eGFP, sub-apical N-Cadherin (pink) based adherens junctions and nuclei (DAPI, blue).

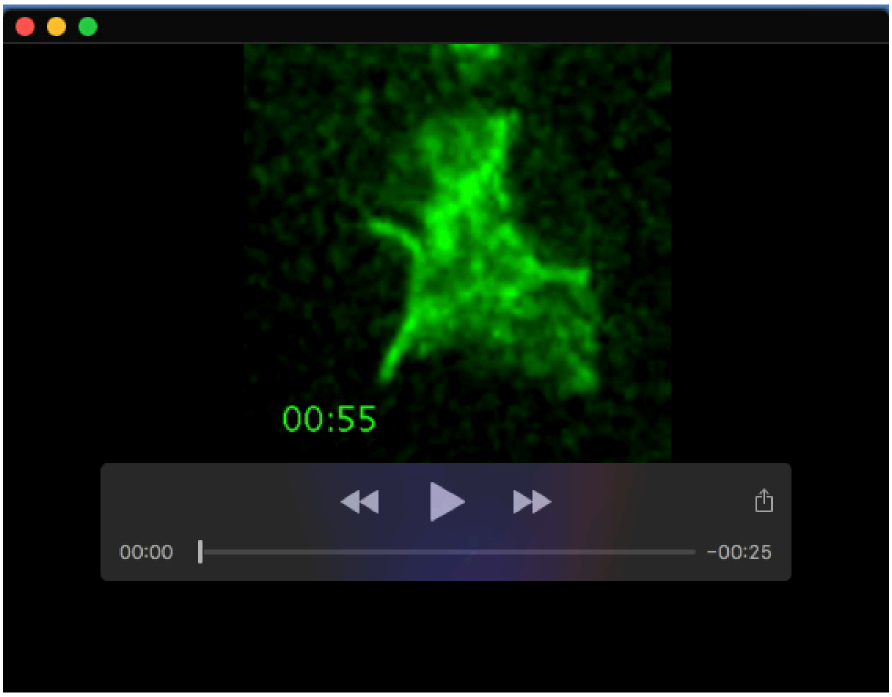

Movie 6. Sub-apical lateral protrusion dynamics. This movie is related to Figure 2F. Mis-expressed pm-eGFP labels the plasma membrane. Maximum intensity projections of deconvolved image sequences were used. Images acquired every 5 seconds.

Journal of Cell Science • Supplementary information

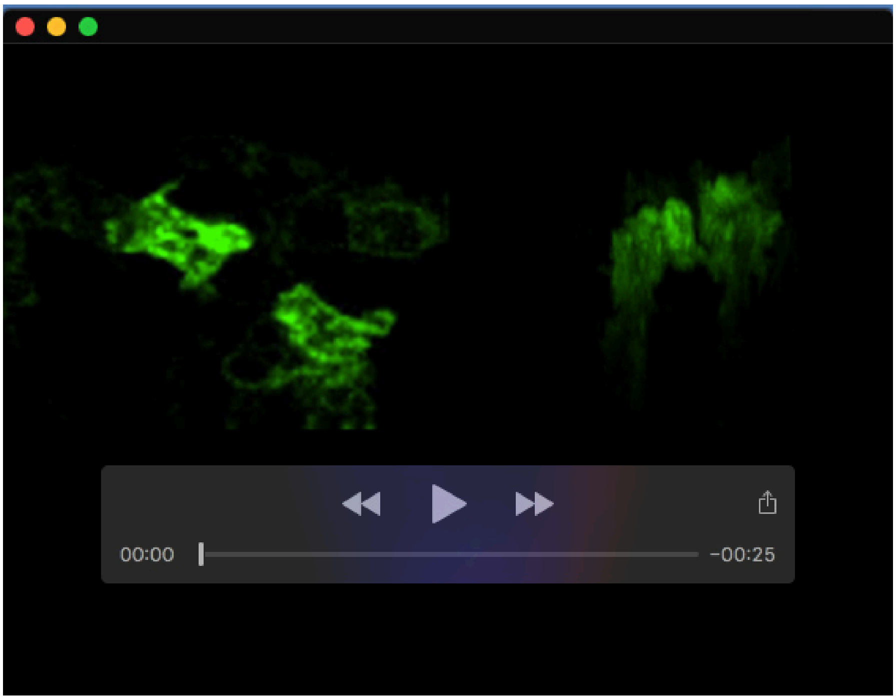

Movie 7. A variety of lateral protrusion dimensions revealed by confocal imaging and 3D models. This movie is related to Supplementary Figure S2A. On the left, apico-basal Z-stack series across the apical plasma membrane and on the right a 3D model projection from the former imaging. Mis-expressed pm-eGFP labels the plasma membrane.

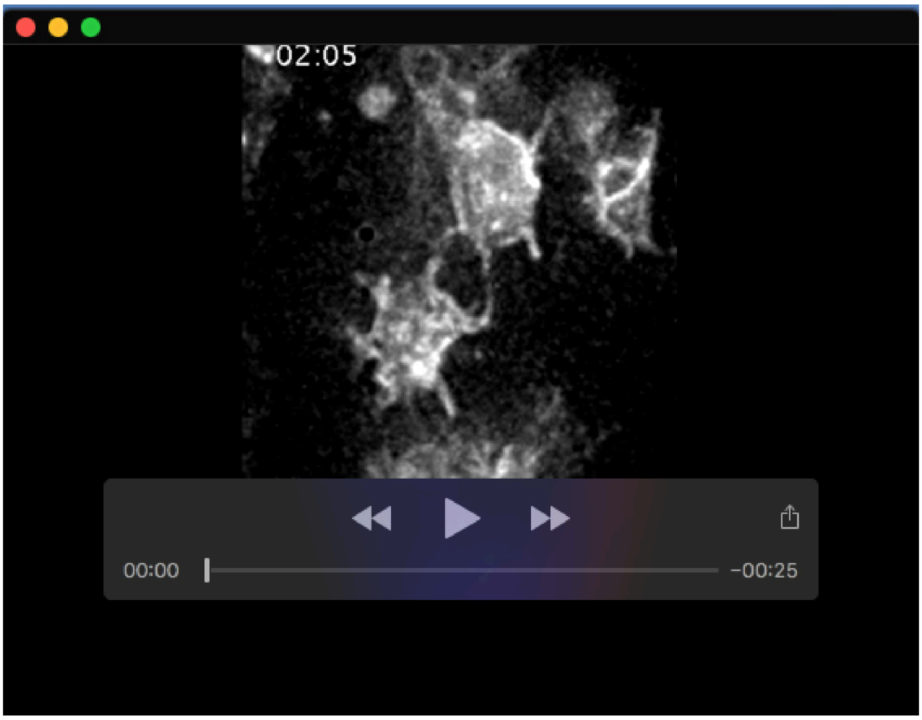

Movie 8. Contact of lateral protrusions from non-neighbouring apical endfeet. These movies are related to Figure 3E. Mis-expressed pm-eGFP labels the plasma membrane. Maximum intensity projections of deconvolved image sequences were used. Images acquired every 5 seconds.

Journal of Cell Science • Supplementary information

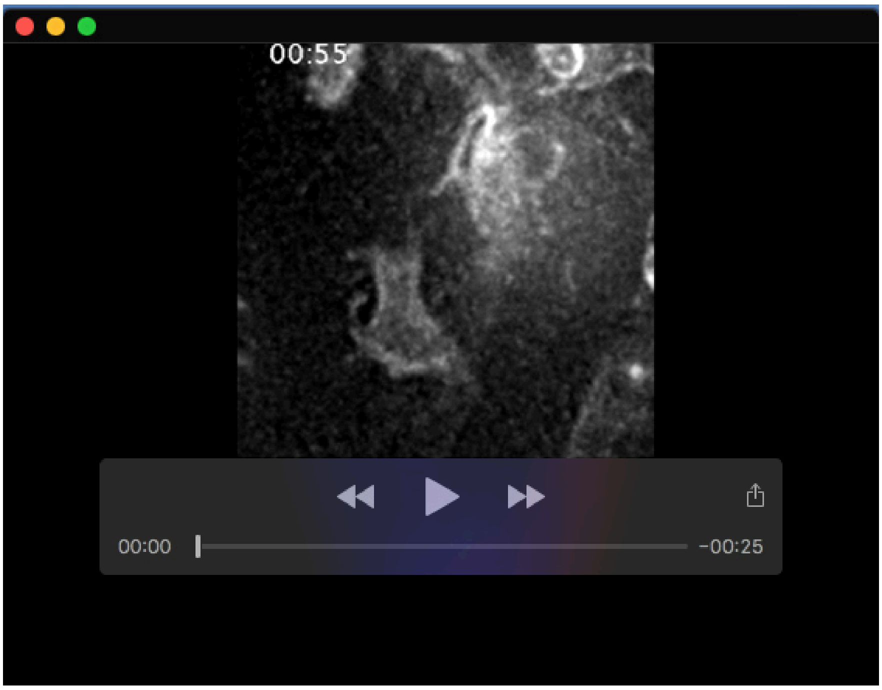

Movie 9. Contact of lateral protrusions from non-neighbouring apical endfeet. These movies are related to Figure 3E. Mis-expressed pm-eGFP labels the plasma membrane. Maximum intensity projections of deconvolved image sequences were used. Images acquired every 5 seconds.

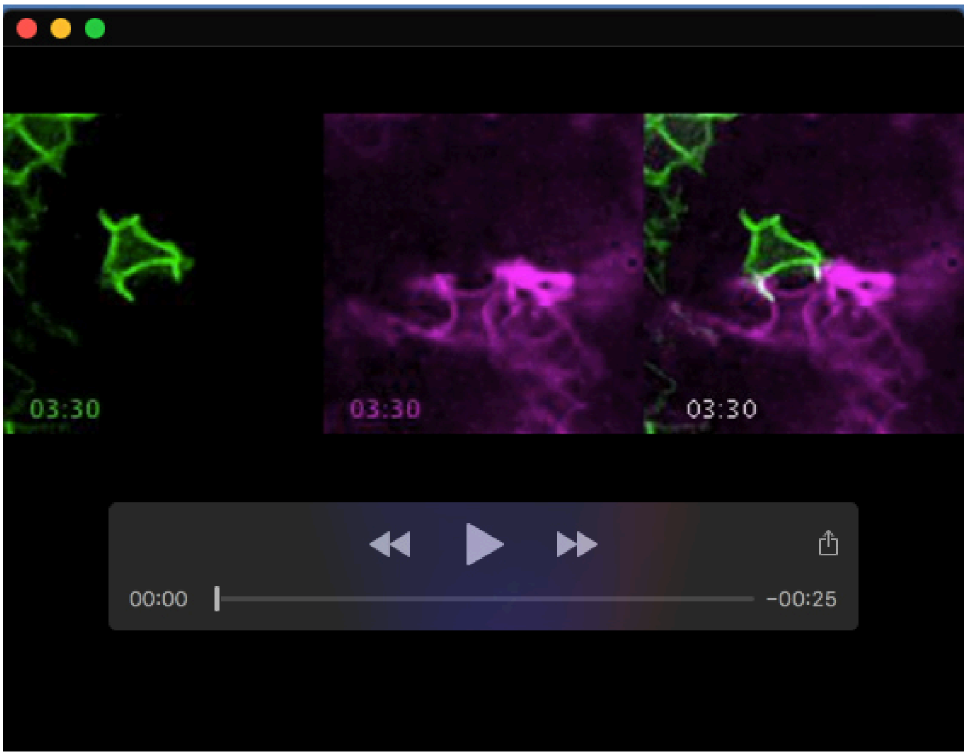

Movie 10. Sub-apical protrusions extended around the plasma membrane of neighbouring endfeet. This movie is related to Figure 4C. Mis-expressed pm-eGFP and pm-mKate2 label the plasma membrane. Maximum intensity projections of deconvolved image sequences were used. Images acquired every 15 seconds.

Journal of Cell Science • Supplementary information

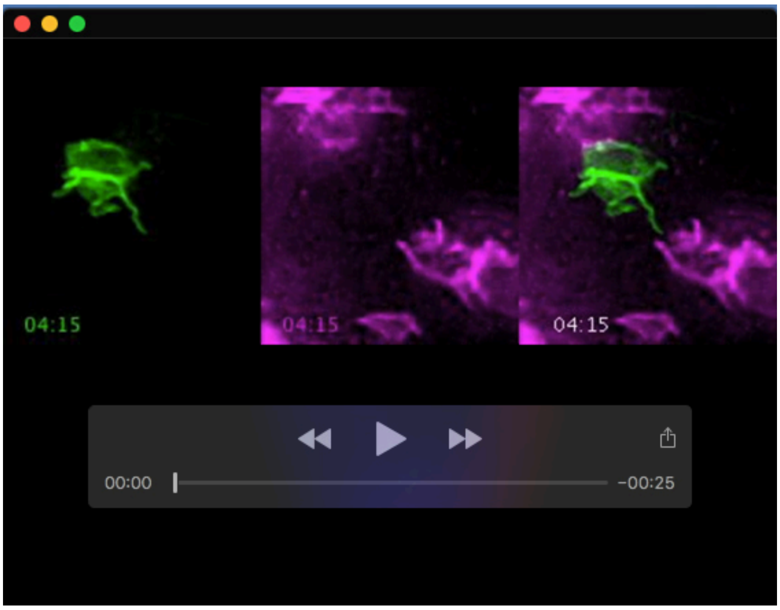

Movie 11. Lateral protrusions from non-neighbouring apical endfeet meet tip to tip and extend along each other. These movies are related to Figures 4D,E. Mis-expressed pm-eGFP and pm-mKate2 label the plasma membrane. Maximum intensity projections of deconvolved image sequences were used. Images acquired every 10 or 15 seconds.

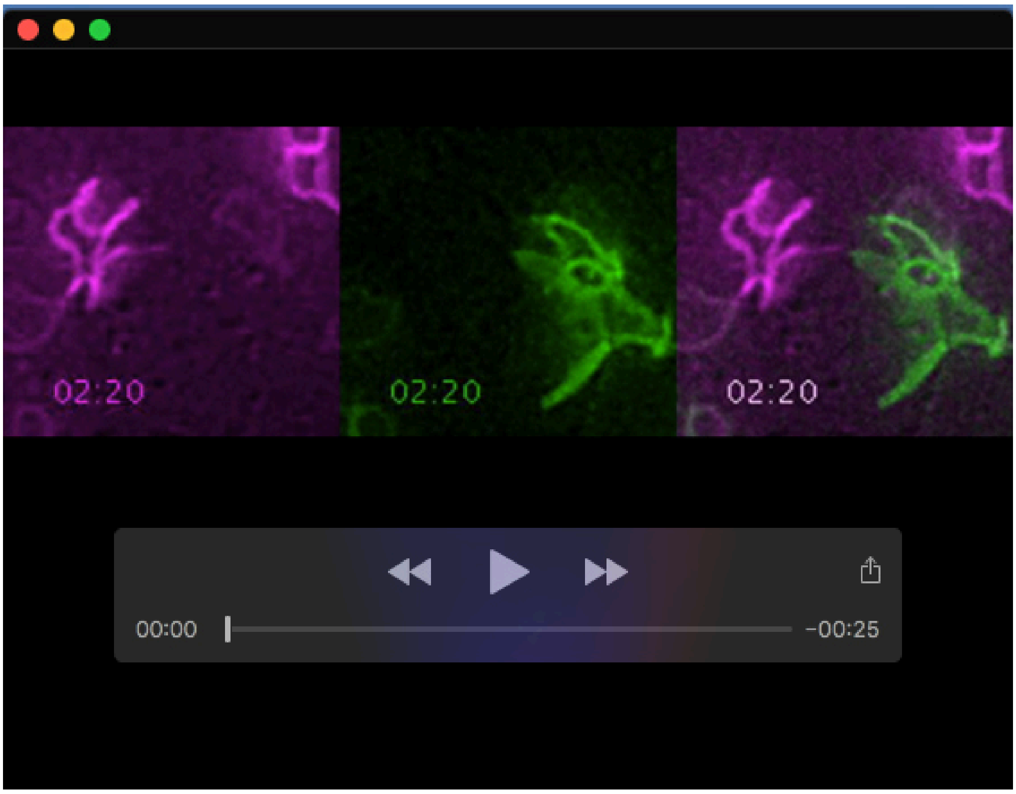

Movie 12. Lateral protrusions from non-neighbouring apical endfeet meet tip to tip and extend along each other. These movies are related to Figures 4D,E. Mis-expressed pm-eGFP and pm-mKate2 label the plasma membrane. Maximum intensity projections of deconvolved image sequences were used. Images acquired every 10 or 15 seconds.

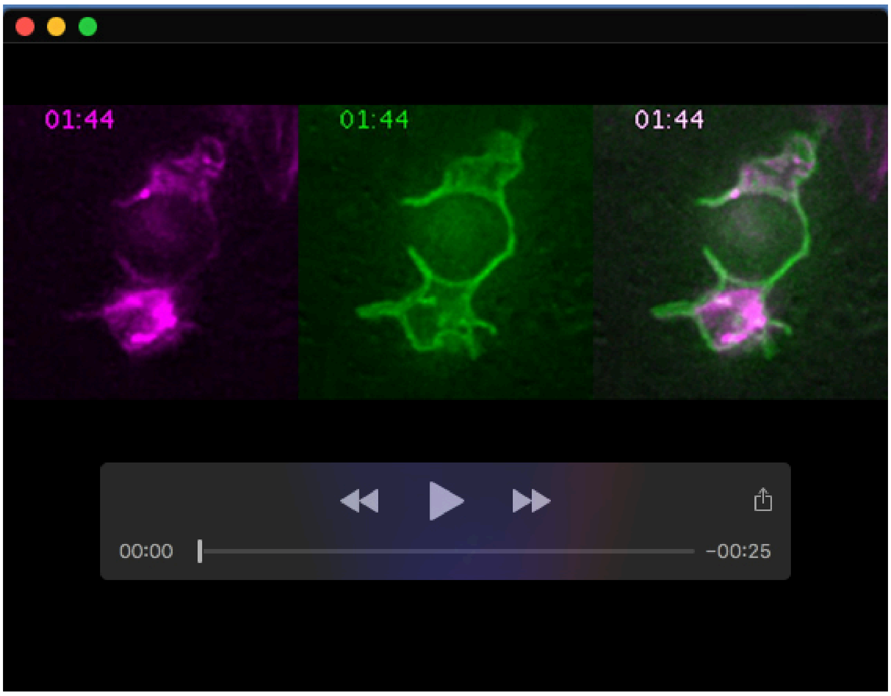

Movie 13. Lateral protrusions contain actin. This movie is related to Figure 5A. Mis-expressed pm-eGFP labels the plasma membrane and F-tractin-mKate2 the actin cytoskeleton. Maximum intensity projections of deconvolved image sequences were used. Images acquired every 8 seconds.

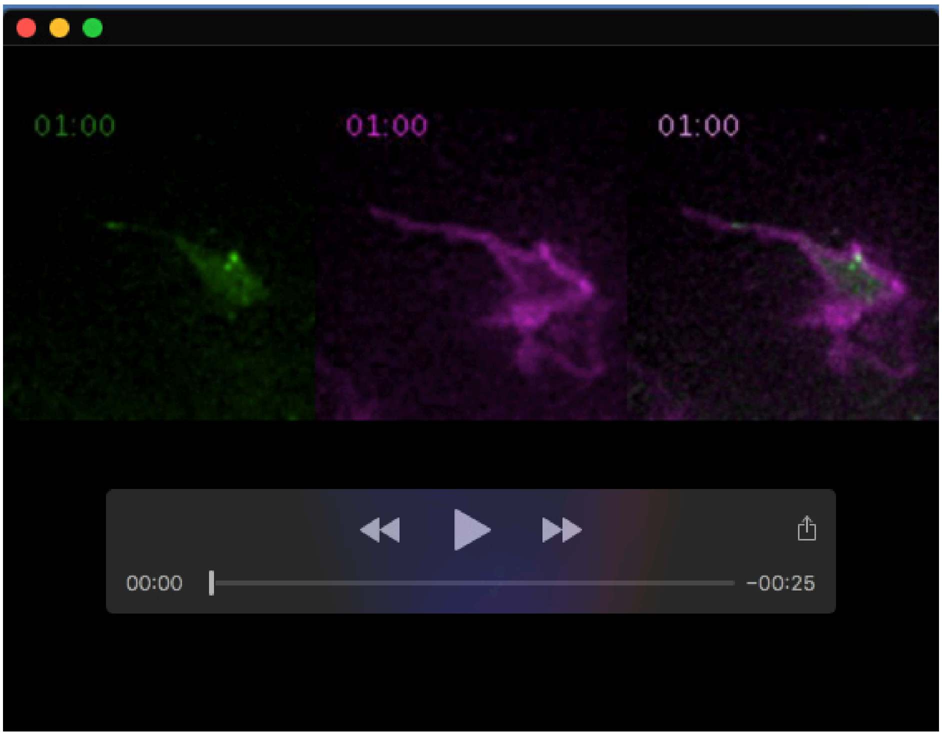

Movie 14. Microtubule growth in lateral protrusions is unidirectional. This movie is related to Figure 5B. Mis-expressed pm-mKate2 labels the plasma membrane and EB3-sfGFP the polymerizing microtubules. Maximum intensity projections of deconvolved image sequences were used. Images acquired every 6 seconds.

Journal of Cell Science • Supplementary information

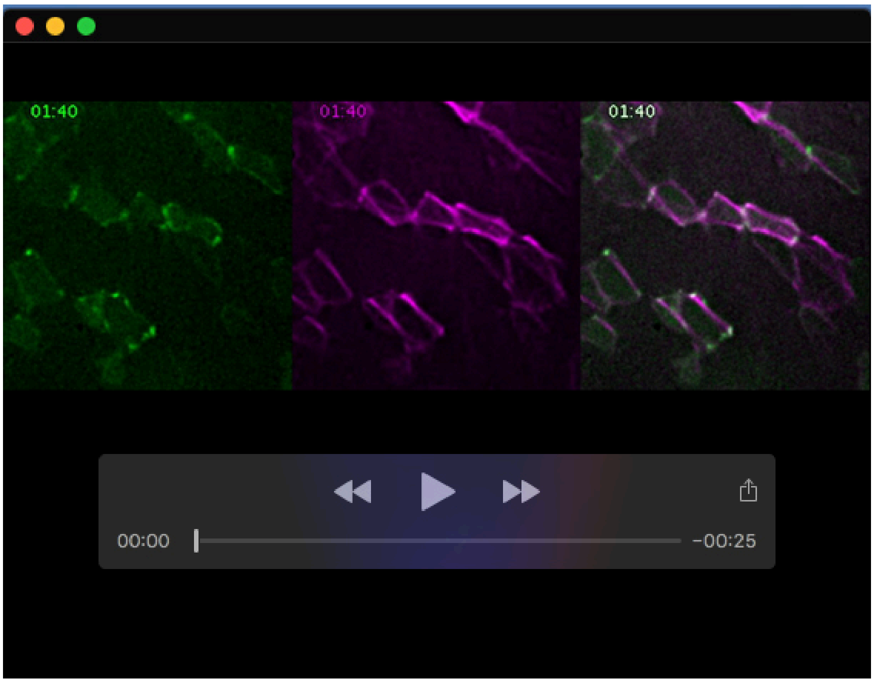

Movie 15. mNeonGreen-ENA localizes at adherens junctions. This movie is related to Figure S2D. Mis-expressed F-tractin-mKate2 labels the actin cytoskeleton. Maximum intensity projections of deconvolved image sequences were used. Images acquired every 10 seconds.

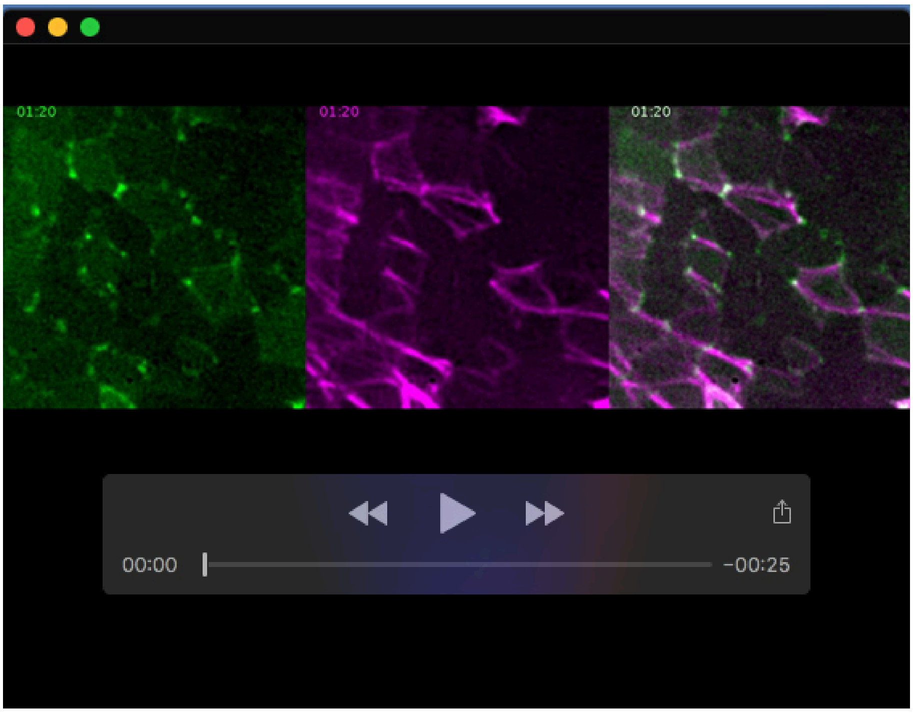

Movie 16. mNeonGreen-VASP localizes at adherens junctions. This movie is related to Figure S2D. Mis-expressed F-tractin-mKate2 labels the actin cytoskeleton. Maximum intensity projections of deconvolved image sequences were used. Images acquired every 10 seconds.

Journal of Cell Science • Supplementary information

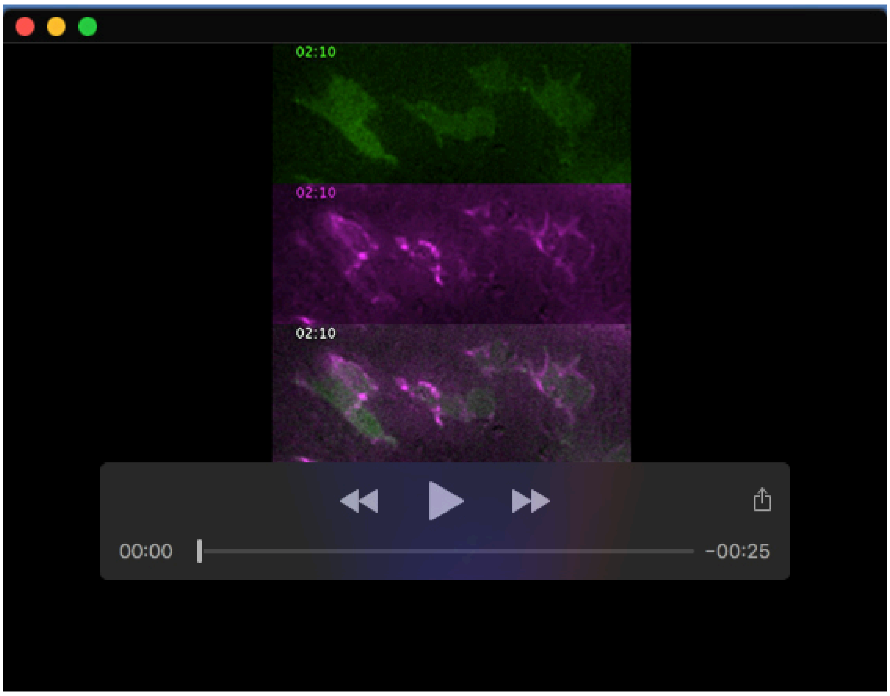

Movie 17. mNeonGreen-ENA localizes at sub-apical protrusions. This movie is related to Figure 5C. Mis-expressed F-tractin-mKate2 labels the actin cytoskeleton. Maximum intensity projections of deconvolved image sequences were used. Images acquired every 10 seconds.

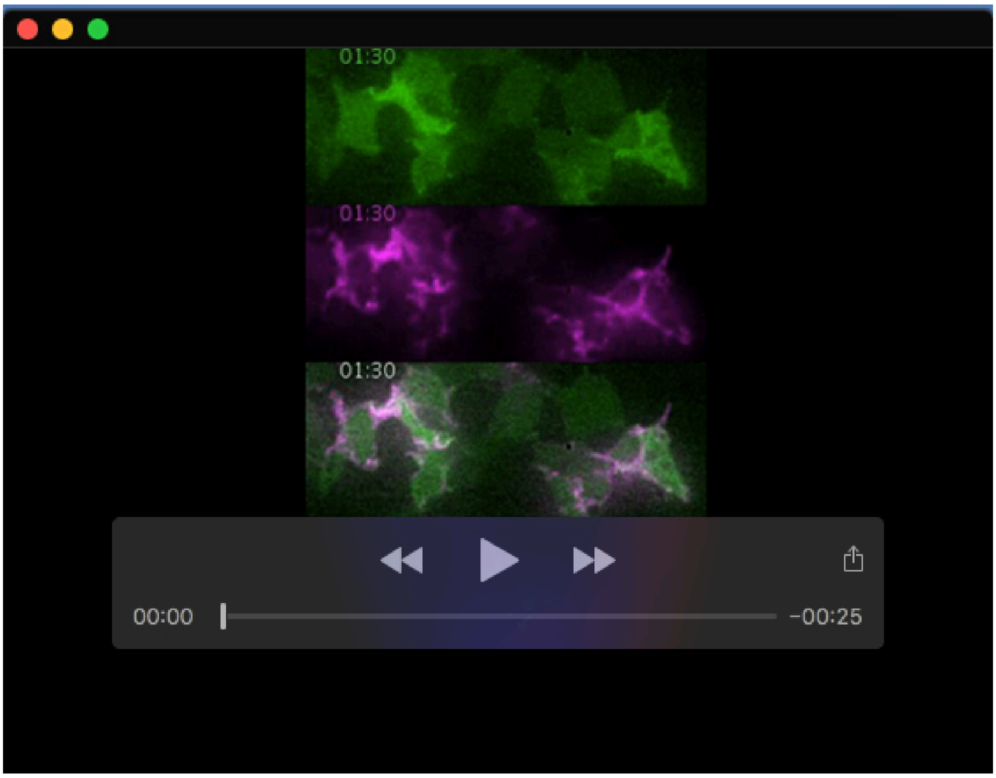

Movie 18. mNeonGreen-VASP localizes at sub-apical protrusions. This movie is related to Figure 5C. Mis-expressed F-tractin-mKate2 labels the actin cytoskeleton. Maximum intensity projections of deconvolved image sequences were used. Images acquired every 10 seconds.

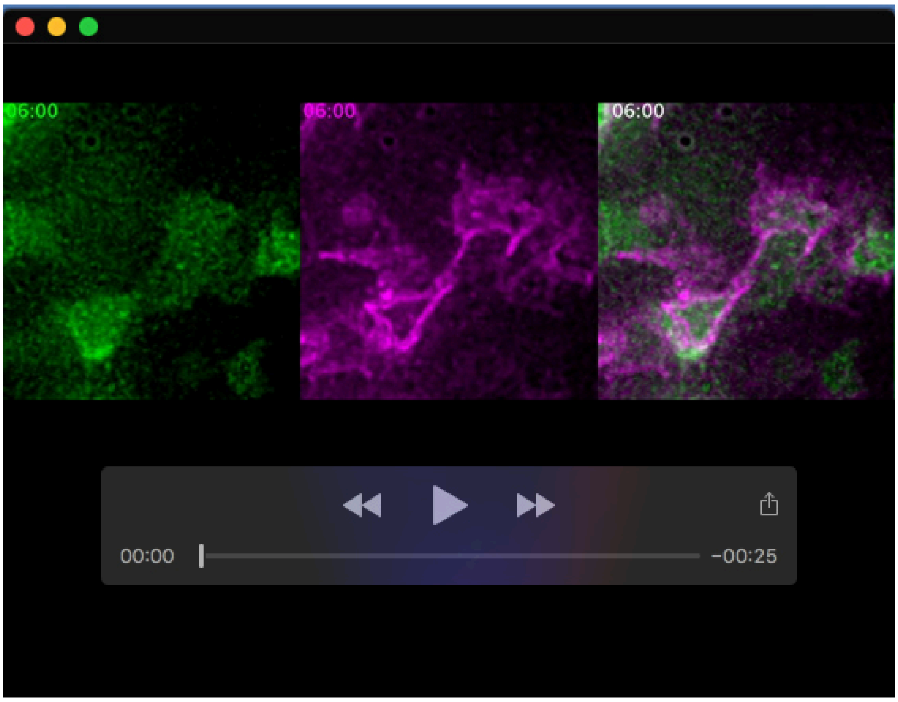

Movie 19. Myosin X (MyoX-eGFP) does not localise to lateral protrusion tips. This movie is related to Supplementary Figure S3F. Mis-expressed pm-mKate2 labels the plasma membrane. Maximum intensity projections of deconvolved image sequences were used. Images acquired every 10 seconds.

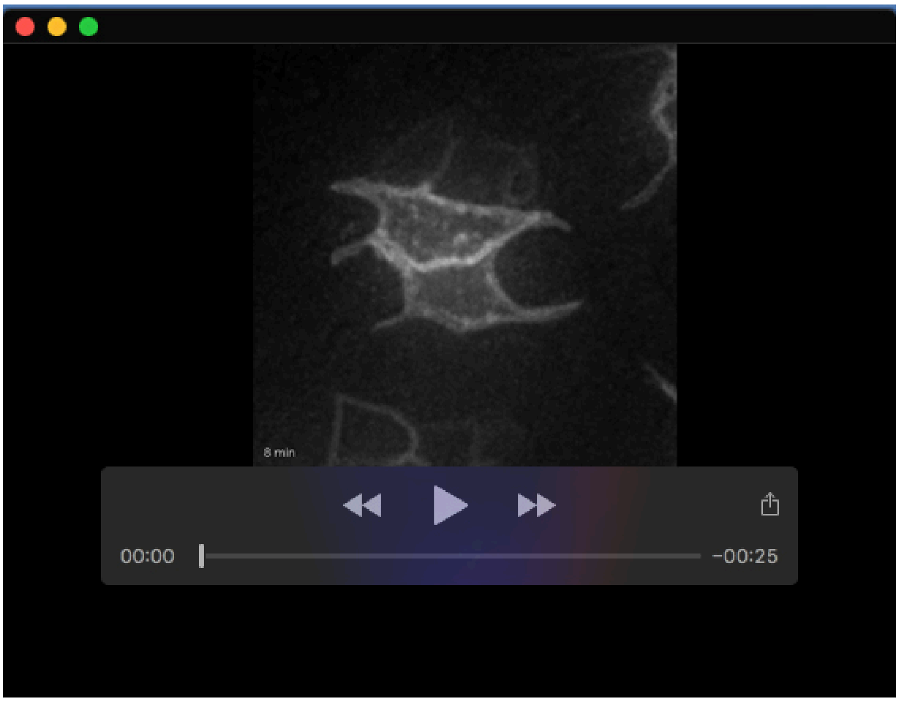

Movie 20. Thin filopodia extend from lateral protrusions. These movies are related to Figure 6A. Mis-expressed pm-eGFP labels the plasma membrane. Maximum intensity projections of deconvolved image sequences were used. Images acquired every 1 minute.

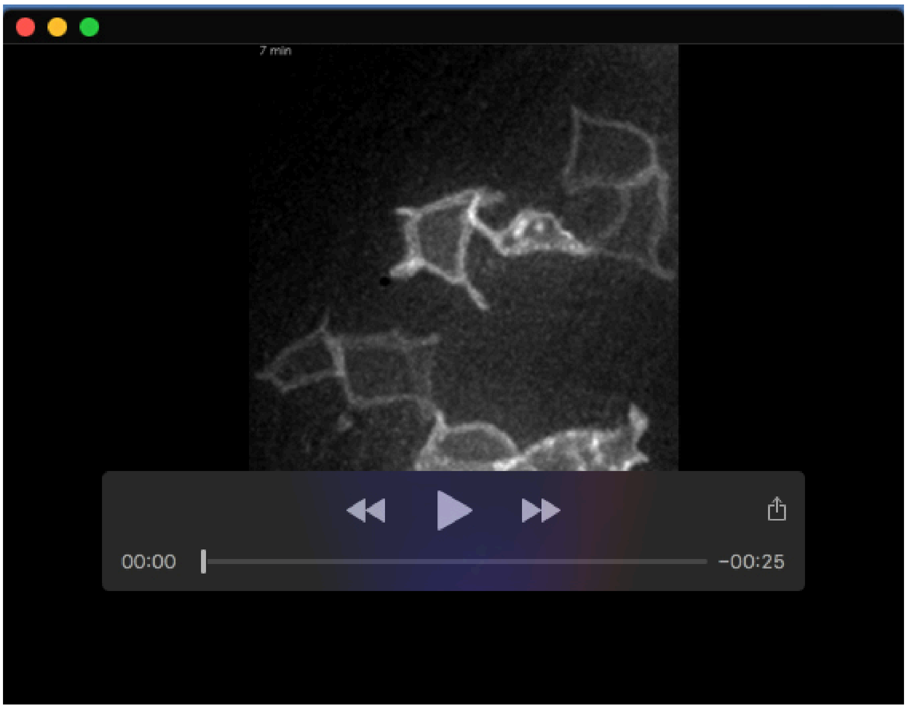

Movie 21. Thin filopodia extend from lateral protrusions. These movies are related to Figure 6A. Mis-expressed pm-eGFP labels the plasma membrane. Maximum intensity projections of deconvolved image sequences were used. Images acquired every 1 minute.

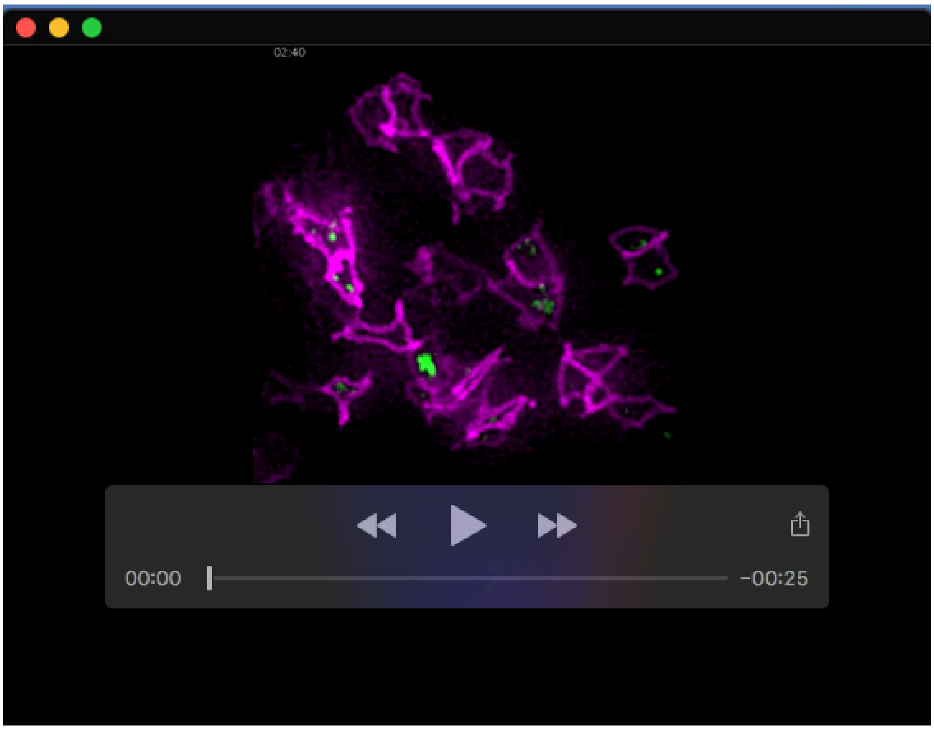

Movie 22. No mitochondrial transfer between cells through the lateral protrusions. This movie is related to Supplementary Figures S3A, B. Mis-expressed mNeonGreen-TOMM20 labels the mitochondria and pm-mKate2 the plasma membrane. Maximum intensity projections of deconvolved image sequences were used. Images acquired every 10 seconds.

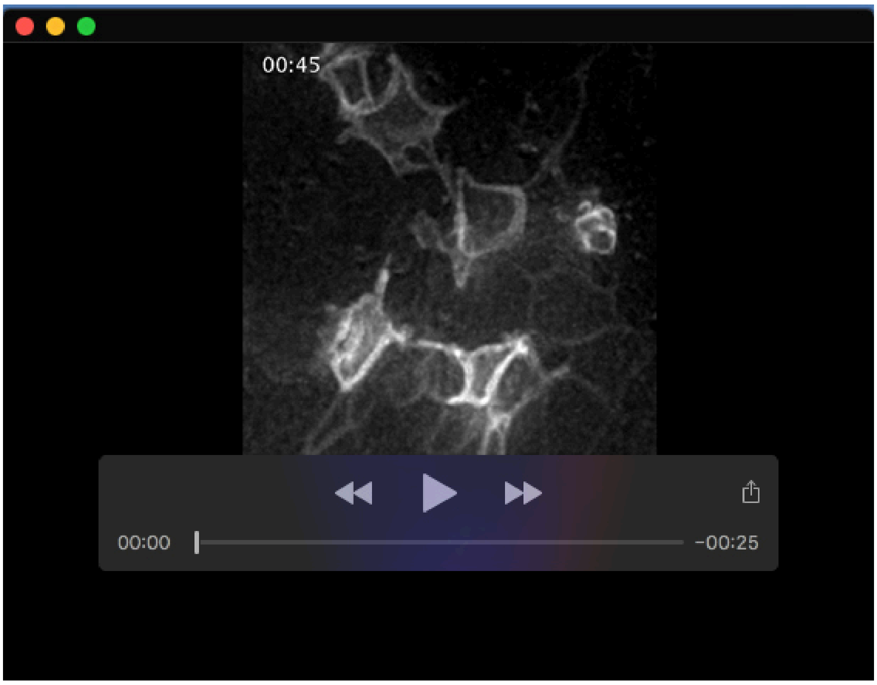

Movie 23. Formation of lateral protrusions depend on actin but not microtubules. These movies are related to Figure 7A (movie 2, DMSO control, movie 24 LatA treated; Movie 25 Taxol treated). Mis-expressed pm-eGFP labels the plasma membrane. Maximum intensity projections of deconvolved image sequences were used. Images acquired every 5 seconds.

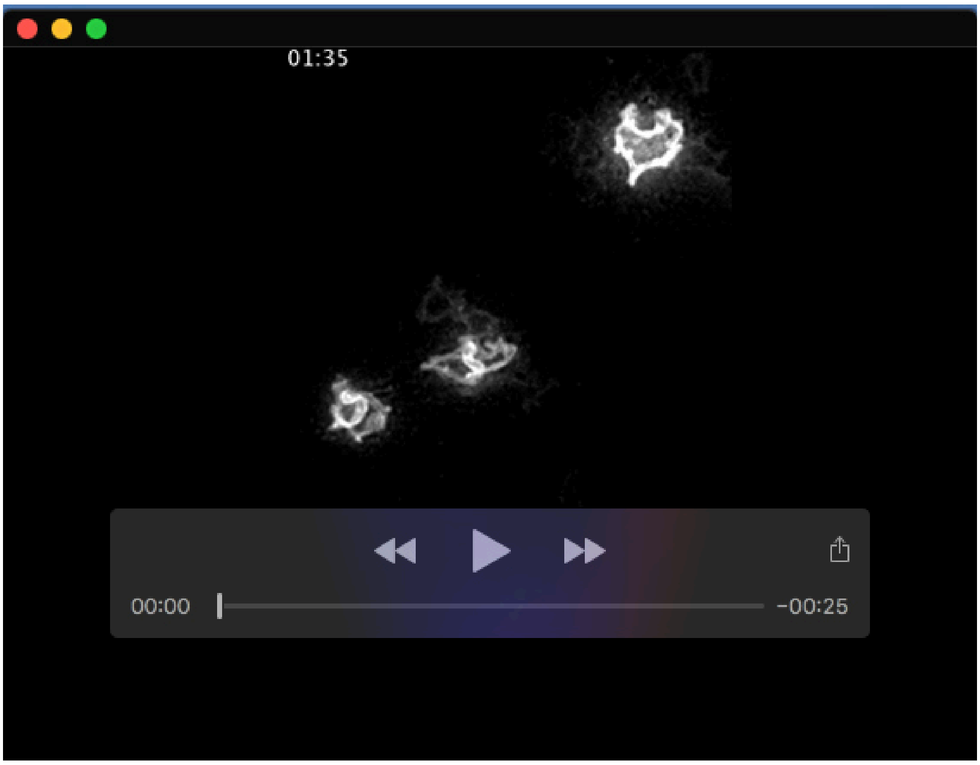

Movie 24. Formation of lateral protrusions depend on actin but not microtubules. These movies are related to Figure 7A (movie 2, DMSO control, movie 24 LatA treated; Movie 25 Taxol treated). Mis-expressed pm-eGFP labels the plasma membrane. Maximum intensity projections of deconvolved image sequences were used. Images acquired every 5 seconds.

Journal of Cell Science • Supplementary information

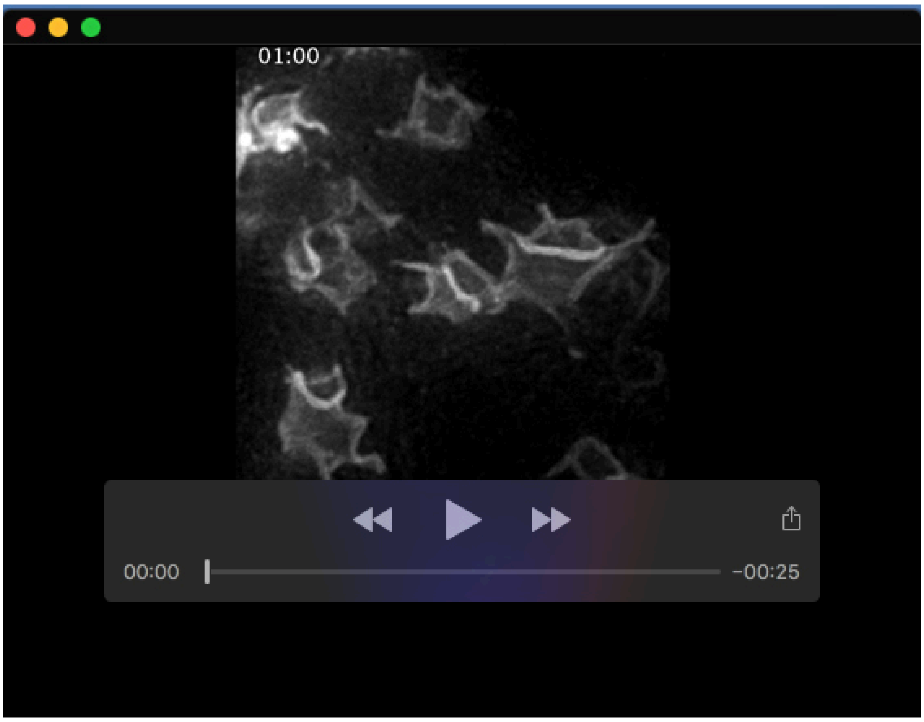

Movie 25. Formation of lateral protrusions depend on actin but not microtubules. These movies are related to Figure 7A (movie 2, DMSO control, movie 24 LatA treated; Movie 25 Taxol treated). Mis-expressed pm-eGFP labels the plasma membrane. Maximum intensity projections of deconvolved image sequences were used. Images acquired every 5 seconds.

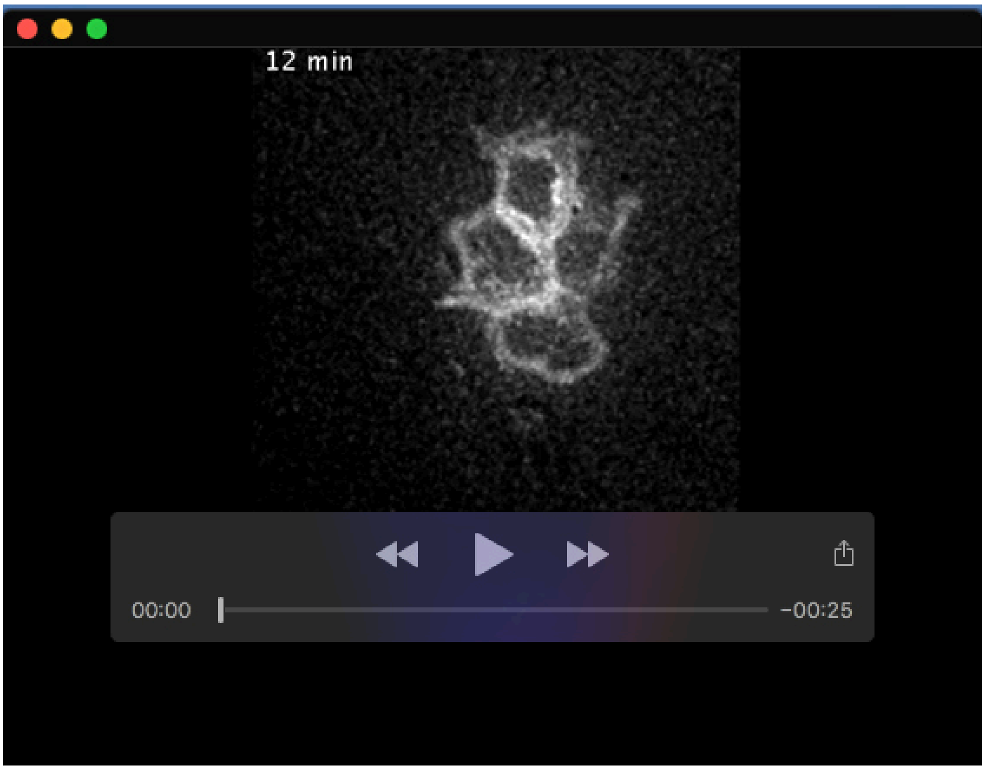

Movie 26. Lateral protrusions following mis-expression of full length WAVE1-eGFP, the latter exhibiting a round ended apical endfoot phenotype. These movies are related to Figure 7E. Mis-expressed pm-mKate2 labels the plasma membrane. Maximum intensity projections of deconvolved image sequences were used. Images acquired every 1 minute.

Journal of Cell Science • Supplementary information

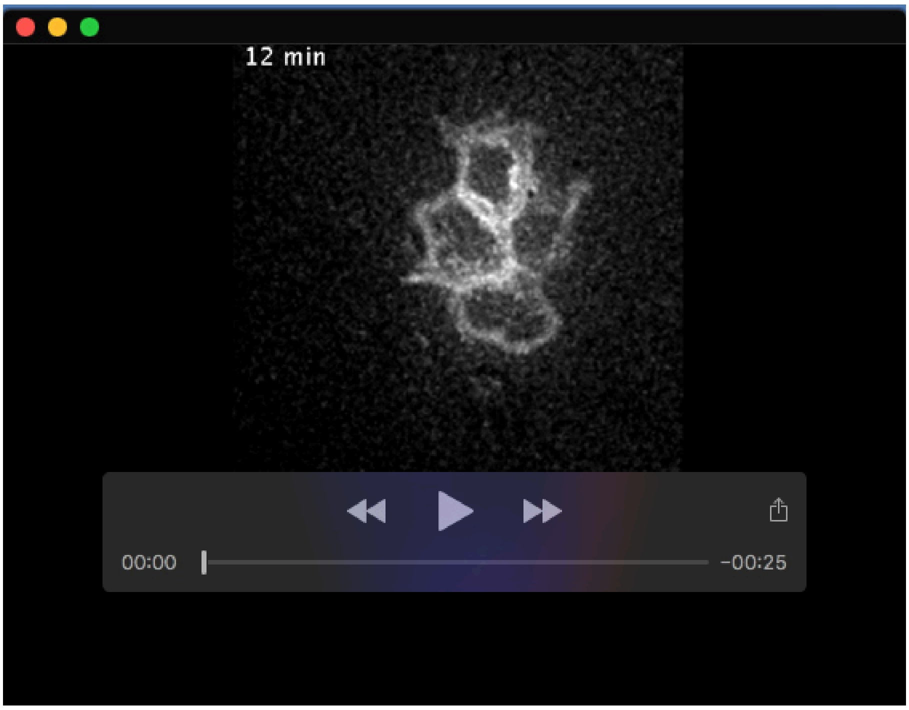

Movie 27. mutant WAVE1-eGFP, the latter exhibiting a round ended apical endfoot phenotype. These movies are related to Figure 7E. Mis-expressed pm-mKate2 labels the plasma membrane. Maximum intensity projections of deconvolved image sequences were used. Images acquired every 1 minute.

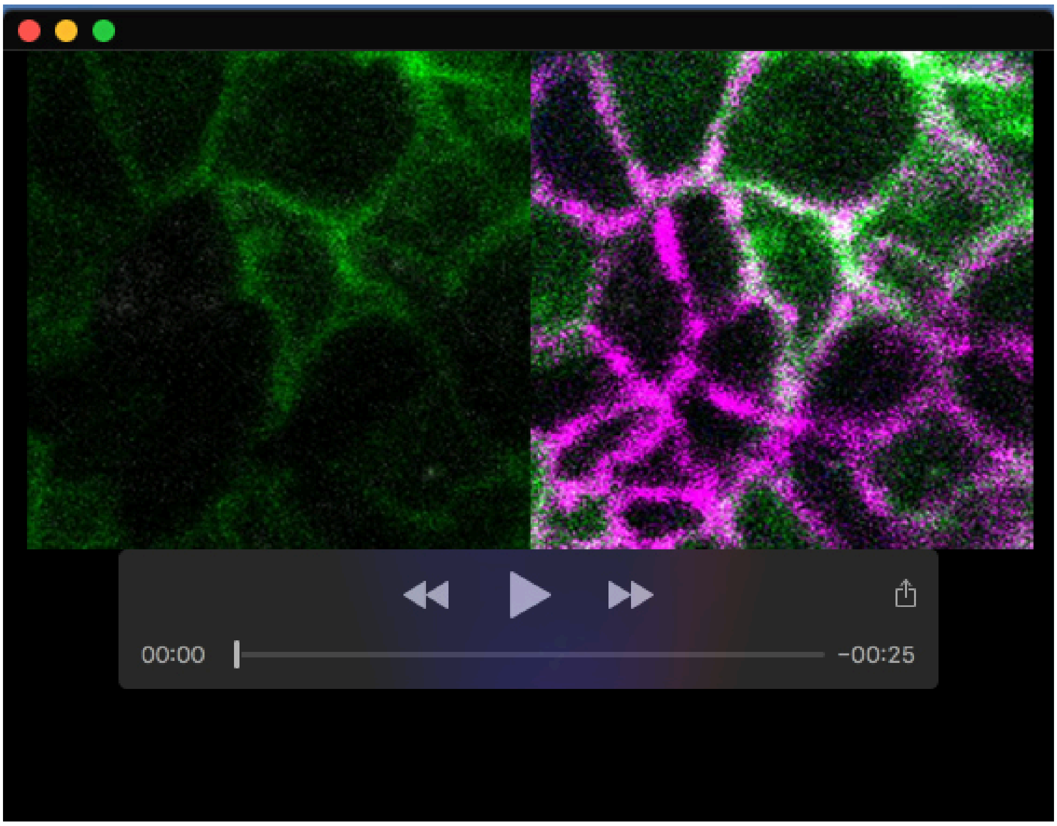

Movie 28. Tuj1 negative progenitors have long lateral protrusions. This movie is related to Figure 8B. Apico-basal Z-stack series across the apical plasma membrane, labelled with pm-eGFP and Tuj1 (left) or pm-eGFP, Tuj1 and N-Cadherin (right).

Journal of Cell Science • Supplementary information

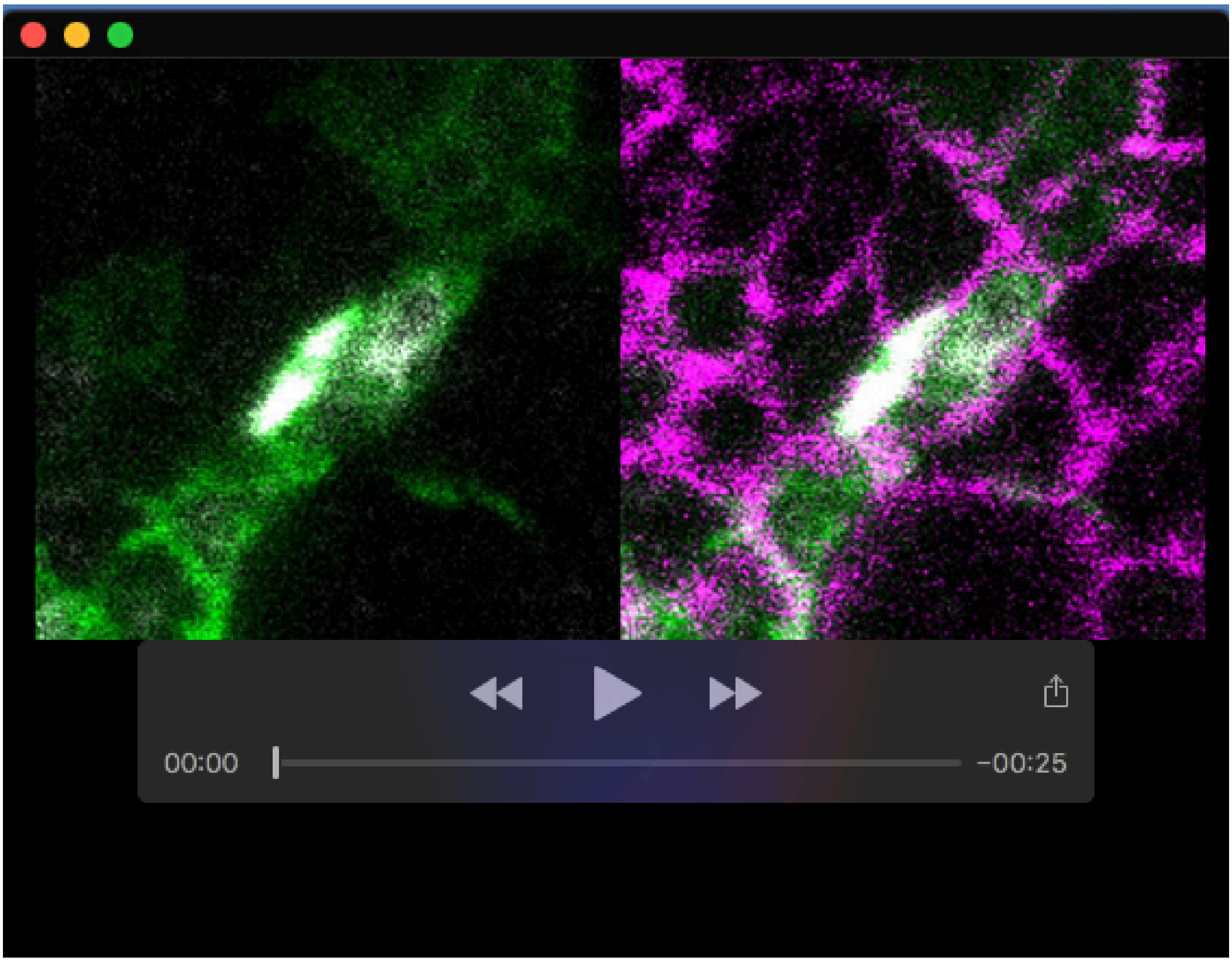

Movie 29. Tuj1 positive neurons have short lateral protrusions. This movie is related to Figure 8B. Apico-basal Z-stack series across the apical plasma membrane, labelled with pm-eGFP and Tuj1 (left) or pm-eGFP, Tuj1 and N-Cadherin (right).
