# Supplementary material for: A lateral protrusion latticework connects neuroepithelial cells and is regulated during neurogenesis
Source: J Cell Sci. 2022 Mar 30;135(6):jcs259897. doi: 10.1242/jcs.259897 (PMC8995095; doi:10.1242/jcs.259897)
Supplement: Reviewer comments [file joces-135-259897_review_history.pdf]

A lateral protrusion latticework connects neuroepithelial cells and is regulated during neurogenesis

Ioannis Kasioulis, Alwyn Dady, John James, Alan R. Prescott, Pamela A. Halley and Kate G. Storey
DOI: 10.1242/jcs.259897

Editor: Michael Way

Review timeline

Submission to Review Commons:	2 November 2021
Original submission:	8 January 2022
Editorial decision:	11 February 2022

Reviewer 1

Evidence, reproducibility and clarity

In this elegant report, Kasioulis and colleagues reenvisage our understanding of neuroepithelial cell morphology. Far removed from the prevalent simplifications of these cells as "wedge shaped" or column-like, they illustrate the co-existence of apical villi, primary cilia, filopodia, lamellipodia, sub-apical protrusions not previously described in the spinal cord, and sub-apical protrusions which appear to extend long filopodia. The sub-apical protrusions are retracted prior to neural differentiation and are diminished in neuroepithelial cells expressing truncated WAVE1.

Major concerns:

1)My understanding is that the authors electroporated truncated WAVE1 mosaically into the neuroepithelium and assessed HUCD positivity in the region of the electroporated cells, finding that the proportion of positively stained cells is comparable to regions of the neural tube without electroporation. The efficiency of electroporation appears variable and low. Given known lateral inhibition of neurogenesis, precocious (or delayed) differentiation of electroporated cells may be compensated by WT cells around them. Please analyse the % GFP+ cells which are HUCD+ and GFP- cells which are HUCD+ on the electroporated side, versus % HUCD+ cells on the non-electroporated side of truncated WAVE1 and GFP-only transfected embryos. Presumably analysis of this data should be paired or use repeated measures statistics (groupings of cells in the same NT). Robustly confirming this negative result would be an important outcome.

2)In Supp Figure 4 and Figure 8E the proportion of GFP-labelled cells is markedly lower in the truncated WAVE1-transfected example than the GFP-only control. Do the authors know if the truncated protein changed construct stability, cell persistence in the NT (apoptosis/extrusion), or whether these images simply reflect variable transfection efficiencies?

3)Do the sub-apical protrusions change predictably during the cell cycle? It seems strange that a cell in S-phase with a basally located nucleus and highly restricted apical domain would have extensive lateral protrusions.

Minor comments:

1) Expression of truncated human WAVE1 in chick cells clearly has quantifiable effects on the apical surface and sub-apical protrusions. It would be reassuring to demonstrate F-actin changes in these cells which could underlie the differences observed, rather than off-target effects such as WAVE interaction with the cell cycle machinery.

© 2022. Published by The Company of Biologists under the terms of the Creative Commons Attribution License (https://creativecommons.org/licenses/by/4.0/).

2) Rather than the subjective "% round-ended phenotype" in figure 7F, the authors could objectively quantify apical cell area, roundness and solidity as commonly used shape descriptors (all standard parameters executable in Fiji).
3) Can the authors use protrusion direction/length vector analysis to determine whether they follow any planar or dorso-ventral polarity which may provide clues to their function?
4) Is apical ZO1 retained in WAVE1 transgenic cells as well as N-cadherin?
5) Please double check WASF/WAVE nomenclature to ensure this is consistent between the results, figure panels and legends.

Significance

The authors provide the first dynamic analysis of a very poorly understood sub-apical cell protrusion in both the chick and human neuroepithelium. The function of these protrusions remains to be established.
These findings will be relevant to epithelial and cell biologists, neural tube and developmental biologists.
My relevant expertise is primarily in neural tube cell biology and application of live imaging methodology used extensively in this manuscript.

Referees cross-commenting

I agree with reviewer 2's assessment of the manuscript and particularly with the comment that it would not be "sensible to ask the authors to hold on to their paper until they uncover a role with more experiments." The novel protrusions they describe are likely to have a range of functions, during neurulation and neurogenesis, the discovery of which will be enabled by the work described here.

Reviewer 2

Evidence, reproducibility and clarity

The paper by Kasioulis et al. describes a previously unknown form of contact between neuroepithelial cells at the apical and sub-apical surface of the chick embryonic spinal cord and human neuroepithelium. For the chicken they use the interlimb area of the neural tube of embryos HH 17-18 and for the human they use fixed embryonic tissue. In the human these contacts are analysed by EM but in the chick neural tube these contacts are additionally characterised by live imaging of electroporated reporters, which results in mosaic expression. Briefly, live imaging identified microvilli, lamellipodia- and filopodia-like protrusions apically and sub-apical lateral protrusions, which are then thoroughly characterised including the speed of 3 types of movement (extension, retraction or lateral) and the protrusion-protrusion contacts. The characterisation of protrusion contacts and their timing is beautiful and a tour de force of live imaging. They further characterise the protrusions molecularly and they show that they contain both actin and MTs but not Myo10 localise, justifying their characterisation as lamelipodia. The authors show that they are not involved in mitochondria transfer so they are distinct from tunnelling nanotubes.

This is the descriptive part of the work and it represents a very thorough analysis. The resulting dynamic description is a strength of the paper. At the same time, the human specimen analysis increases the relevance of the findings to human developmental biology and this is also a strength of the paper.

The functional part of the paper relies on the use of a truncated (i.e. dominant negative) actin binding protein WAVE 1. The authors disrupt the formation of protrusions with this DN WAVE-1 protein as a tool (ΔWASF1-eGFP mis-expression), and they observe that there was no disruption of AJs, tissue integrity or the rate of neurogenesis. Thus, the functional experiments present mostly negative results. It is possible that there is an effect in some other cellular property, perhaps in the organisation of signalling. In fact, this is very likely to be the case.

There are no major issues affecting the conclusions. The weakness of the paper lies in this functional part in that a role for these protrusions has not been uncovered. However, I don't think that it would be sensible to ask the authors to hold on to their paper until they uncover a role with

© 2022. Published by The Company of Biologists under the terms of the Creative Commons Attribution License (https://creativecommons.org/licenses/by/4.0/).

more experiments. It is very likely that the paper will be very useful to others who study the role of signalling in the organisation of neurogenesis in neural tube and as such, I believe that it will be very useful to other research and I predict that it will highly cited.

Minor points

1) The text is a little contradictory as to how far these protrusions go to connect cells: For example in line 107 we are told that they "extend over several endfoot diameters" (also at line 543) but elsewhere, such as in the diagram it says "1-2 endfoot diameters" which agrees with the data summarised in lines 231-232. I assume that neighbours further than the immediate neighbours are only reached by the combined length of the filopodia and lamelipodia and only transiently. Perhaps a more careful textual description here is needed to eliminate any inconsistencies.

2) Line 114: "a role for the regulation of the WAVE1-mediated actin network in neuronal delamination". A role of WAVE-1 in lateral protrusions is indeed shown, and the cells that do delaminate downregulate their lateral protrusions but where is it shown that DN-WAVE1 affects delamination? The text may need to be appropriately adjusted.

3) Having said that, in Figure 8E DWAVE1-sGFP panel, it looks like more GFP cells are located basally than in the pm-eGFP control (even though there is no increase in the number of cells expressing HuC/D). Can this be clarified please? Is this a real result?

4) Figure 1C "Focussing on contacts between neuroepithelial cells we identified thin plasma membrane that spread between adjacent endfeet (black arrow heads Figure 1C) apical to adherens junctions (white arrowheads)" I am sorry but I really cant see anything where the black arrowhead points, including the high magnification image. Can this be shown more clearly? Same with the yellow arrowheads in Figure 1A, although Figure 1D is more convincing with respect to the yellow arrowheads.

5) The analysis of expression of the Notch ligand Delta1 expression pattern in WAVE1 DN electroporated embryos is said to be normal but it is presented as data not shown. Some journals no longer accept "data not shown" since either the data is important and done well enough to be shown or they should be taken out of the paper. With this in mind, I think the data should either be shown or removed from the paper (lines 433-435).

Significance

The paper focuses mostly on the sub-apical protrusions which are the least understood, although a previous study has described them in the mouse cortex in Shinoda et al., 2018, but not with live imaging. These lateral protrusions appear in all neuroepithelial cells and therefore the authors suggest that they form a latticework of interactions that are short range but extend beyond the immediate neighbours.

Thus, the key message of the paper is that these protrusions extend the contact that cells make to non-adjacent cells. The evidence for this is convincing. The authors offer mainly two non-mutually exclusive scenarios to explain their findings, that it, either the phenomenon is part of the mechanism that anchors cells apically in the neuroepithelium and its loss is an early event in delamination (but by itself not sufficient to trigger delamination) or, the lamellipodia are involved in some sort of cell-cell signalling of unknown function at present.

The value of the paper is that it shows that neuroepithelial cells make cells beyond their immediate neighbours, potentially via these lamellipodia described here; this may have an impact in cell-to-cell signalling, such as local co-ordination of some signalling activity, perhaps Notch-Delta signalling. As they correctly say this will necessitate establishing the potential localisation of ligands and receptors in these protrusions but this would be substantial extra work and I think it is out of the scope of this paper. Here, it may useful to add a reference to a paper that shows how powerful signalling through protrusions is in generating different patterns https://doi.org/10.1098/rsif.2016.0484

© 2022. Published by The Company of Biologists under the terms of the Creative Commons Attribution License (https://creativecommons.org/licenses/by/4.0/).

The paper will be of great interest to developmental and cell biologists. It will be particularly useful to those seeking to understand signalling in the neural tube and the organisation of the neurogenesis as an emergent property of the interaction of single cells. By showing that cells interact beyond their immediate neighbours it provides an essential piece of information, even if it is not known yet what this interaction means.

My expertise is in vertebrate neurogenesis, transcription factors, Notch-Delta signalling and live imaging.

Referees cross-commenting

I agree with the reviewers 1's assessment. Addressing the comments will improve the manuscript. My only concern is that the connection with the cell cycle may not be easy to show directly as it would involve imaging on a different plane. However, they may be able to infer some relationship from the size of the apical domain. I am sure the authors will carefully consider whether this is something that they can address with confidence.

Author response to reviewers' comments

Review Commons Reviews for Refereed Preprint RC-2021-01138

Reviewer #1 (Evidence, reproducibility and clarity (Required)):
In this elegant report, Kasioulis and colleagues reenvisage our understanding of neuroepithelial cell morphology. Far removed from the prevalent simplifications of these cells as "wedge shaped" or column-like, they illustrate the co-existence of apical villi, primary cilia, filopodia, lamellipodia, sub-apical protrusions not previously described in the spinal cord, and sub-apical protrusions which appear to extend long filopodia. The sub-apical protrusions are retracted prior to neural differentiation and are diminished in neuroepithelial cells expressing truncated WAVE1.

Major concerns:

1) My understanding is that the authors electroporated truncated WAVE1 mosaically into the neuroepithelium and assessed HUC/D positivity in the region of the electroporated cells, finding that the proportion of positively stained cells is comparable to regions of the neural tube without electroporation. The efficiency of electroporation appears variable and low. Given known lateral inhibition of neurogenesis, precocious (or delayed) differentiation of electroporated cells may be compensated by WT cells around them. Please analyse the % GFP+ cells which are HUC/D+ and GFP- cells which are HUC/D+ on the electroporated side, versus % HUC/D+ cells on the non-electroporated side of truncated WAVE1 and GFP-only transfected embryos. Presumably analysis of this data should be paired or use repeated measures statistics (groupings of cells in the same NT). Robustly confirming this negative result would be an important outcome.

To address this, we determined the % GFP+ cells which are HuC/D+ and % GFP- cells which are HuC/D+ on the electroporated side of all embryos. We then performed paired t-tests comparing %HuC/D+/GFP+ and %HuC/D+/GFP- within each embryo for each of the two conditions (truncated) ΔWAVE1-eGFP and control pm-eGFP. In the control and the truncated WAVE1 conditions we found a statistically significant difference between eGFP expressing and non-eGFP expressing cells ($p = < 0.0001$ and $p = 0.0017$ respectively), while an unpaired t-test showed that there was no significant difference ($p = 0.3838$) between the two conditions. This indicates that a subset of cells is transfected in both these contexts and shows that introduction of the ΔWAVE1-eGFP does not affect neuronal differentiation any more than the control construct. To further control for the potential effect of electroporation we also compared % of cells expressing HuC/D on electroporated and non-electroporated sides within each embryo/section for the two conditions and this revealed no statistical difference ($p = 0.3281$). We have revised the text and refer to this additional analysis which is presented in new Supplementary Figure 6. With this further analysis we robustly confirm this negative result.

© 2022. Published by The Company of Biologists under the terms of the Creative Commons Attribution License (https://creativecommons.org/licenses/by/4.0/).

The apparent increased basal localization of cell bodies in Figure 8E for the ΔWAVE1-eGFP construct may reflect the cytoplasmic localization of this protein in comparison with membrane localization of pm-GFP and we have now noted this in the figure legend.

2) In Supp Figure 4 and Figure 8E the proportion of GFP-labelled cells is markedly lower in the truncated WAVE1-transfected example than the GFP-only control. Do the authors know if the truncated protein changed construct stability, cell persistence in the NT (apoptosis/extrusion), or whether these images simply reflect variable transfection efficiencies?

The GFP only control is more easily expressed in most electroporation experiments. It was easy to select embryos electroporated with the GFP-only or ΔWAVE1-eGFP 24hs post electroporation, however, by 48h this was more difficult for ΔWAVE1-eGFP. This might reflect reduced stability of the truncated protein. We found no evidence of cell death (apoptotic figures in DAPI staining) and nor cell extrusion (cell debris in the neural tube lumen).

3) Do the sub-apical protrusions change predictably during the cell cycle? It seems strange that a cell in S-phase with a basally located nucleus and highly restricted apical domain would have extensive lateral protrusions.

This is an interesting point. We do not think it is strange that a cell is S-phase with a basally located nucleus has lateral protrusions, it may for example, be signalling to others that it has entered the cell cycle and/or have received signals promoting this via these protrusions. In our quantifications of protrusion parameters, we analysed structures in the first 5µm from the apical surface in the apical endfeet of all cells, except those where the nucleus was at the apical surface (this included cells in mitosis and potentially late G2 and early G1). We did not systematically analyse lateral protrusion parameters in cells in different cell cycle phases - indeed this would require labelling individual cells with cell cycle phase specific markers and a membrane marker, but we did assess cells with TUJ1 expression (which are in G1) and have basally located nuclei. These presumptive neurons had a smaller apical area and shorter lateral protrusions, consistent with withdrawal of these structures prior to neuronal delamination. We can provide further data showing that lateral protrusions persist even as cells transit mitosis. We include this in the text now (line 197) and provide an image in supplementary data and note in the text that lateral protrusions were found extending from all apical endfeet examined. Our observations suggest that these structures persist through the cell cycle.

Minor comments:

1)Expression of truncated human WAVE1 in chick cells clearly has quantifiable effects on the apical surface and sub-apical protrusions. It would be reassuring to demonstrate F-actin changes in these cells which could underlie the differences observed, rather than off-target effects such as WAVE interaction with the cell cycle machinery.

Our expression studies with WAVE1 constructs indicate that this protein localizes to the base of lateral protrusions and so to a subset of F-actin. We show that ΔWAVE1-eGFP leads to reduction/loss of lateral protrusions, which contain F-actin, so it is possible that in the presence of ΔWAVE1-eGFP, when the protrusions are lost, F-actin is no longer detected at these focal points. We are unclear how our results could be explained by off-target effects on the cell cycle, for example, we found no evidence for arrest in mitosis following mis-expression of ΔWAVE1-eGFP at 24 or 48h. If this is deemed an essential revision, we will undertake this new analysis.

2) Rather than the subjective "% round-ended phenotype" in figure 7F, the authors could objectively quantify apical cell area, roundness and solidity as commonly used shape descriptors (all standard parameters executable in Fiji).

We have now performed these quantifications in ImageJ for apical cell area, circularity, solidity and roundness and then used unpaired t-tests to compare between full-length and truncated WAVE1. Cells expressing truncated WAVE1 had a smaller apical endfoot area ($p < 0.0001$) and shorter perimeter ($p < 0.0001$) and had greater circularity and solidity (both comparisons $p < 0.0001$), than cells expressing FL- WAVE1, while roundness did not differ ($p=0.2288$). For these comparisons, FL-WAVE1: 2 experiments, 4 explants, 120 cells; truncated WAVE1: 4 experiments, 9

© 2022. Published by The Company of Biologists under the terms of the Creative Commons Attribution License (https://creativecommons.org/licenses/by/4.0/).

explants, 146 cells). These further quantifications are included in the revised paper (Lines 373-376) and new figure 6G.

3) Can the authors use protrusion direction/length vector analysis to determine whether they follow any planar or dorso-ventral polarity which may provide clues to their function?

This is an interesting question. However, this would require extensive analysis and potentially further data generation to draw firm conclusions. We feel this is beyond the scope of this paper.

4) Is apical ZO1 retained in WAVE1 transgenic cells as well as N-cadherin?

This would be nice to know as it represents a further apical membrane marker, but the result would not alter the main findings of the paper. We think this is not essential.

5) Please double check WASF/WAVE nomenclature to ensure this is consistent between the results, figure panels and legends.

Apologies, this has been addressed. We retain WASF1/WAVE1 where we refer to previous research using WASF1 nomenclature and use WAVE1 in all other contexts now.

Reviewer #1 (Significance (Required)):

The authors provide the first dynamic analysis of a very poorly understood sub-apical cell protrusion in both the chick and human neuroepithelium. The function of these protrusions remains to be established.
These findings will be relevant to epithelial and cell biologists, neural tube and developmental biologists.

My relevant expertise is primarily in neural tube cell biology and application of live imaging methodology used extensively in this manuscript.

Referees cross-commenting

I agree with reviewer 2's assessment of the manuscript and particularly with the comment that it would not be "sensible to ask the authors to hold on to their paper until they uncover a role with more experiments." The novel protrusions they describe are likely to have a range of functions, during neurulation and neurogenesis, the discovery of which will be enabled by the work described here.

Reviewer #2 (Evidence, reproducibility and clarity (Required)):

The paper by Kasioulis et al. describes a previously unknown form of contact between neuroepithelial cells at the apical and sub-apical surface of the chick embryonic spinal cord and human neuroepithelium. For the chicken they use the interlimb area of the neural tube of embryos HH 17-18 and for the human they use fixed embryonic tissue. In the human these contacts are analysed by EM but in the chick neural tube these contacts are additionally characterised by live imaging of electroporated reporters, which results in mosaic expression. Briefly, live imaging identified microvilli, lamellipodia- and filopodia-like protrusions apically and sub-apical lateral protrusions, which are then thoroughly characterised including the speed of 3 types of movement (extension, retraction or lateral) and the protrusion-protrusion contacts. The characterisation of protrusion contacts and their timing is beautiful and a tour de force of live imaging. They further characterise the protrusions molecularly and they show that they contain both actin and MTs but not Myo10 localise, justifying their characterisation as lamelipodia. The authors show that they are not involved in mitochondria transfer so they are distinct from tunnelling nanotubes.

This is the descriptive part of the work and it represents a very thorough analysis. The resulting dynamic description is a strength of the paper. At the same time, the human specimen analysis increases the relevance of the findings to human developmental biology and this is also a strength

© 2022. Published by The Company of Biologists under the terms of the Creative Commons Attribution License (https://creativecommons.org/licenses/by/4.0/).

of the paper.

The functional part of the paper relies on the use of a truncated (i.e. dominant negative) actin binding protein WAVE 1. The authors disrupt the formation of protrusions with this DN WAVE-1 protein as a tool (ΔWASF1-eGFP mis-expression), and they observe that there was no disruption of AJs, tissue integrity or the rate of neurogenesis. Thus, the functional experiments present mostly negative results. It is possible that there is an effect in some other cellular property, perhaps in the organisation of signalling. In fact, this is very likely to be the case.

There are no major issues affecting the conclusions. The weakness of the paper lies in this functional part in that a role for these protrusions has not been uncovered. However, I don't think that it would be sensible to ask the authors to hold on to their paper until they uncover a role with more experiments. It is very likely that the paper will be very useful to others who study the role of signalling in the organisation of neurogenesis in neural tube and as such, I believe that it will be very useful to other research and I predict that it will highly cited.

Minor points

1) The text is a little contradictory as to how far these protrusions go to connect cells: For example in line 107 we are told that they "extend over several endfoot diameters" (also at line 543) but elsewhere, such as in the diagram it says "1-2 endfoot diameters" which agrees with the data summarised in lines 231-232. I assume that neighbours further than the immediate neighbours are only reached by the combined length of the filopodia and lamelipodia and only transiently. Perhaps a more careful textual description here is needed to eliminate any inconsistencies.

We have now clarified the extent of lateral protrusions throughout the MS (see tracked changes). This apparent discrepancy arose because we begin by considering lateral protrusions without their filopodial extension. The schematic summary provided in figure 9 is correct. These structures extend around their immediate neighbours and contact protrusions from non-neighbouring cells at this point. With the filopodial extensions they can further contact the endfeet of cells beyond their immediate neighbours (as well as the earlier contact with protrusions from non-immediate neighbours) - so in total lateral protrusions and their filopodia can extend up to several (=2) endfoot diameters.

2) Line 114: "a role for the regulation of the WAVE1-mediated actin network in neuronal delamination". A role of WAVE-1 in lateral protrusions is indeed shown, and the cells that do delaminate downregulate their lateral protrusions but where is it shown that DN-WAVE1 affects delamination? The text may need to be appropriately adjusted.

This sentence refers to these findings, lateral protrusions are withdrawn prior to delamination, loss of WAVE1 function reduces lateral protrusions, we therefore think that this implicates that regulation of WAVE1 as early step that prefigures delamination (noted in the Discussion). In this sentence (line 114) we say “identifies a role …” we have changed this now to “implicate regulation of WAVE1 …”

3)Having said that, in Figure 8E DWAVE1-sGFP panel, it looks like more GFP cells are located basally than in the pm-eGFP control (even though there is no increase in the number of cells expressing HuC/D). Can this be clarified please? Is this a real result?

This has been addressed above in our reply to the first comment from reviewer 1.

4)Figure 1C «Focussing on contacts between neuroepithelial cells we identified thin plasma membrane that spread between adjacent endfeet (black arrow heads Figure 1C) apical to adherens junctions (white arrowheads)" I am sorry but I really cant see anything where the black arrowhead points, including the high magnification image. Can this be shown more clearly? Same with the yellow arrowheads in Figure 1A, although Figure 1D is more convincing with respect to the yellow arrowheads.

We have revised Figure 1C, these images have been enlarged and we have used a purple overlay to indicate the plasma membrane spread between adjacent endfeet. We note that such lamellipodia

© 2022. Published by The Company of Biologists under the terms of the Creative Commons Attribution License (https://creativecommons.org/licenses/by/4.0/).

structures are also demonstrated in our live imaging data, presented in Figure 2A and parameterized in Figure 2B. We have also enlarged and better indicated with a purple overlay for the protrusion in Figure 1A. We can provide the non-overlay images as supplementary data. Please also note that all the figures are low res png files inserted into the text for the ease in initial review.

5) The analysis of expression of the Notch ligand Delta1 expression pattern in WAVE1 DN electroporated embryos is said to be normal but it is presented as data not shown. Some journals no longer accept "data not shown" since either the data is important and done well enough to be shown or they should be taken out of the paper. With this in mind, I think the data should either be shown or removed from the paper (lines 433-435).

This data and images showing that the pattern of *Delta1* expression in embryos expressing ΔWAVE1-eGFP is unchanged can be provided as supplementary data. We see no difference in the pattern of *Delta1* expression and control pmGFP and ΔWAVE1-eGFP expressing neural tube (> 6 sections of neural tube from n=2 control and n=3 ΔWAVE1-eGFP expressing embryos).

Reviewer #2 (Significance (Required)):

The paper focuses mostly on the sub-apical protrusions which are the least understood, although a previous study has described them in the mouse cortex in Shinoda et al., 2018, but not with live imaging. These lateral protrusions appear in all neuroepithelial cells and therefore the authors suggest that they form a latticework of interactions that are short range but extend beyond the immediate neighbours.

Thus, the key message of the paper is that these protrusions extend the contact that cells make to non-adjacent cells. The evidence for this is convincing. The authors offer mainly two non-mutually exclusive scenarios to explain their findings, that it, either the phenomenon is part of the mechanism that anchors cells apically in the neuroepithelium and its loss is an early event in delamination (but by itself not sufficient to trigger delamination) or, the lamellipodia are involved in some sort of cell-cell signalling of unknown function at present.

The value of the paper is that it shows that neuroepithelial cells make cells beyond their immediate neighbours, potentially via these lamellipodia described here; this may have an impact in cell-to-cell signalling, such as local co-ordination of some signalling activity, perhaps Notch-Delta signalling. As they correctly say this will necessitate establishing the potential localisation of ligands and receptors in these protrusions but this would be substantial extra work and I think it is out of the scope of this paper. Here, it may useful to add a reference to a paper that shows how powerful signalling through protrusions is in generating different patterns https://doi.org/10.1098/rsif.2016.0484

This is indeed an interesting paper which models how cellular protrusions mediating Notch/Delta signalling could regulate pattern. We now cite this earlier publication alongside Hadjivasiliou et al 2019.

The paper will be of great interest to developmental and cell biologists. It will be particularly useful to those seeking to understand signalling in the neural tube and the organisation of the neurogenesis as an emergent property of the interaction of single cells. By showing that cells interact beyond their immediate neighbours it provides an essential piece of information, even if it is not known yet what this interaction means.

My expertise is in vertebrate neurogenesis, transcription factors, Notch-Delta signalling and live imaging.

Referees cross-commenting

I agree with the reviewers 1's assessment. Addressing the comments will improve the manuscript. My only concern is that the connection with the cell cycle may not be easy to show directly as it would involve imaging on a different plane. However, they may be able to infer some relationship

© 2022. Published by The Company of Biologists under the terms of the Creative Commons Attribution License (https://creativecommons.org/licenses/by/4.0/).

from the size of the apical domain. I am sure the authors will carefully consider whether this is something that they can address with confidence.

Original submission

First decision letter

MS ID#: JOCES/2022/259897

MS TITLE: A lateral protrusion latticework connects neuroepithelial cells and is regulated during neurogenesis

AUTHORS: Ioannis Kasioulis, Alwyn Dady, John James, Alan R Prescott, Pamela A Halley, and Kate G Storey
ARTICLE TYPE: Research Article

Thank you for sending your manuscript to Journal of Cell Science through Review Commons.

I am happy to tell you that your manuscript has been accepted for publication in Journal of Cell Science, pending standard ethics checks.

© 2022. Published by The Company of Biologists under the terms of the Creative Commons Attribution License (https://creativecommons.org/licenses/by/4.0/).
